# Genetic Landscape of Major Depressive Disorder: Assessment of Potential Diagnostic and Antidepressant Response Markers

**DOI:** 10.1093/ijnp/pyad001

**Published:** 2023-01-19

**Authors:** Priyanka Singh, Ankit Srivastava, Debleena Guin, Sarita Thakran, Jyoti Yadav, Puneet Chandna, Mamta Sood, Rakesh Kumar Chadda, Ritushree Kukreti

**Affiliations:** Genomics and Molecular Medicine Unit, Council of Scientific and Industrial Research (CSIR) - Institute of Genomics and Integrative Biology (IGIB), New Delhi, India; Academy of Scientific and Innovative Research (AcSIR), Ghaziabad, India; Genomics and Molecular Medicine Unit, Council of Scientific and Industrial Research (CSIR) - Institute of Genomics and Integrative Biology (IGIB), New Delhi, India; Department of Pharmacology, School of Pharmaceutical Education and Research, Jamia Hamdard, New Delhi, India; Genomics and Molecular Medicine Unit, Council of Scientific and Industrial Research (CSIR) - Institute of Genomics and Integrative Biology (IGIB), New Delhi, India; Department of Biotechnology, Delhi Technological University, Shahbad Daulatpur, Delhi, India; Genomics and Molecular Medicine Unit, Council of Scientific and Industrial Research (CSIR) - Institute of Genomics and Integrative Biology (IGIB), New Delhi, India; Academy of Scientific and Innovative Research (AcSIR), Ghaziabad, India; Genomics and Molecular Medicine Unit, Council of Scientific and Industrial Research (CSIR) - Institute of Genomics and Integrative Biology (IGIB), New Delhi, India; Indian Society of Colposcopy and Cervical Pathology (ISCCP), Safdarjung Hospital, New Delhi, India; Department of Psychiatry, All India Institute of Medical Sciences, Ansari Nagar, New Delhi, India; Department of Psychiatry, All India Institute of Medical Sciences, Ansari Nagar, New Delhi, India; Genomics and Molecular Medicine Unit, Council of Scientific and Industrial Research (CSIR) - Institute of Genomics and Integrative Biology (IGIB), New Delhi, India; Academy of Scientific and Innovative Research (AcSIR), Ghaziabad, India

**Keywords:** Major depressive disorder, antidepressants response, genetic association studies, diagnostic predictability, pharmacogenetics

## Abstract

**Background:**

The clinical heterogeneity in major depressive disorder (MDD), variable treatment response, and conflicting findings limit the ability of genomics toward the discovery of evidence-based diagnosis and treatment regimen. This study attempts to curate all genetic association findings to evaluate potential variants for clinical translation.

**Methods:**

We systematically reviewed all candidates and genome-wide association studies for both MDD susceptibility and antidepressant response, independently, using MEDLINE, particularly to identify replicated findings. These variants were evaluated for functional consequences using different in silico tools and further estimated their diagnostic predictability by calculating positive predictive values.

**Results:**

A total of 217 significantly associated studies comprising 1200 variants across 545 genes and 128 studies including 921 variants across 412 genes were included with MDD susceptibility and antidepressant response, respectively. Although the majority of associations were confirmed by a single study, we identified 31 and 18 replicated variants (in at least 2 studies) for MDD and antidepressant response. Functional annotation of these 31 variants predicted 20% coding variants as deleterious/damaging and 80.6% variants with regulatory effect. Similarly, the response-related 18 variants revealed 25% coding variant as damaging and 88.2% with substantial regulatory potential. Finally, we could calculate the diagnostic predictability of 19 and 5 variants whose positive predictive values ranges from 0.49 to 0.66 for MDD and 0.36 to 0.66 for response.

**Conclusions:**

The replicated variants presented in our data are promising for disease diagnosis and improved response outcomes. Although these quantitative assessment measures are solely directive of available observational evidence, robust homogenous validation studies are required to strengthen these variants for molecular diagnostic application.

**Significance Statement**

Our work provides a holistic landscape of all genetic association studies related to major depressive disorder (MDD) risk and antidepressant response. In consideration of the well-established fact that several genetic association investigations have identified variants underlying disease susceptibility and antidepressant response outcome, no biomarker is available to date for clinical application. Therefore, our prime objective was to evaluate the accumulating genetic findings to unearth the clinically important genetic markers. Hence, we curated all the existing literature of genetic association studies related to MDD susceptibility and antidepressant response. Additionally, we examined functional relevance and diagnostic predictability of the replicated variants. Altogether, our work proposed some potential candidate variants from the literature that are likely to be utilized for clinical translation in MDD following an appropriate validation.

## INTRODUCTION

Major depressive disorder (MDD) is a common, complex, and debilitating neuropsychiatric disorder that contributes a significant burden on both the individual and society (Norkeviciene et al., 2022). It is primarily characterized by symptoms that include low mood, anhedonia, fatigue, alteration in sleep and appetite, lack of concentration, feelings of guilt, helplessness, and in the worst cases, suicidal ideations (Malhi and Mann, 2018). According to the WHO, nearly 4.4% of the world population are affected by depression, with a significant rate of suicidal deaths of approximately 1 million per year (World Health Organization, 2017).

To date, the diagnosis of MDD revolves around the symptom-based approach, which often becomes difficult due to the lack of defined natural boundaries and overlapping symptoms between affective disorders (Feczko et al., 2019; García-Gutiérrez et al., 2020). Furthermore, the treatment of depression involves a trial-and-error approach, wherein monotherapy initially is favored followed by multi-drug therapy in case patients fail to respond to initial therapy. Nevertheless, one-third of the patients do not adequately respond to the existing pharmacotherapy regimen. This variability could be a consequence of intrinsic biological (inter-individual genetic variations) and environmental heterogeneity among MDD patients (Fabbri et al., 2019; Srivastava et al., 2019). Therefore, it is imperative to identify certain bio-signatures (such as single nucleotide polymorphisms (SNPs)) associated with the MDD susceptibility and antidepressant response, enabling the appropriate diagnosis and promoting personalized medication/therapy.

The etiology of MDD is multifactorial, involving a complex interaction of both genetic and environmental factors. The genetic contribution to pathophysiology has been demonstrated by a plethora of genetic association studies in addition to twin studies that reported the heritability of MDD to be approximately 40% (Sullivan et al., 2000; Shadrina et al., 2018; Schwabe et al., 2019). The existing literature regarding the genetics of MDD and treatment response is enormous and complex. The 2 complementary approaches primarily exploited in genetic studies are genome-wide association studies (GWAS) and candidate gene studies. Until recently, the majority of candidate studies primarily focused on genes related to a widely accepted hypothesis based on a serotonergic, hypothalamic-pituitary-adrenal (HPA) axis, neural plasticity, glutamatergic system considering their role in MDD pathophysiology and the fact that they might be the targets of antidepressant drugs (Basu et al., 2015, 2019). Most of them have reported a positive association with the disease susceptibility and treatment response despite that low power of the study and contradictory findings limit their clinical applicability. Moreover, a recent study by Border et al. (2019) on a large sample pool examined the association of 18 highly studied candidate gene polymorphisms with depression and found no substantial contribution of any of the polymorphisms to depression liability. On the other hand, earlier GWAS studies failed to detect SNPs of a genome-wide significance level (Lewis et al., 2010; Muglia et al., 2010; Rietschel et al., 2010; Shi et al., 2011), but recent GWAS and meta-analyses have revealed 15 (Hyde et al., 2016), 16 (Howard et al., 2021), 44 (Wray et al., 2018), and 102 (Howard et al., 2019) genetic loci to be significantly associated with MDD susceptibility. However, these results across studies were not consistently reproducible. These inconsistencies likely may be explained by methodological differences such as the definition of depression and sampling strategies employed. We speculate positive association in a single study can be argued as false positive; thus, reproducible associations hold promise and would have tremendous importance in prognosis, diagnosis, and therapeutics.

Therefore, in the present study, we systematically collated the available genetic association data and identified the replicated genetic variants and performed their functional annotation and diagnostic predictability analysis in MDD susceptibility and antidepressant response, independently, via a PubMed-based search strategy. Our work is an attempt to find if any genetic variant holds the potential for clinical translation based on the available observational evidence.

## METHODS

A systematic search was performed using MEDLINE database wherein we independently retrieved the literature for all genetic association studies related to MDD susceptibility and antidepressant response.

### Search Strategy and Study Selection

#### Acquisition of MDD Risk–Associated Studies—

A comprehensive PubMed search (https://pubmed.ncbi.nlm.nih.gov/) was conducted using a combination of the following medical subject heading terms “major depressive disorder”, “major depression”, “depressive disorder”, “genetics”, “gene”, “SNP”, “polymorphism”, and “variants” with AND/OR Boolean operators to retrieve relevant publications until June 2021. Articles were manually screened for titles, abstracts, and full texts by 2 authors (P.S. and A.S.) independently. Any discrepancies were resolved on consensus agreement with a third author (R.K.). Two authors (D.G. and S.T.) cross-checked the data to ensure consistency. Additionally, the bibliography of included articles was subsequently screened for additional references. The inclusion criteria were as follows: (1) patients primarily diagnosed as MDD; (2) a case-control study-design; (3) the study must examine the association between a variant/s and MDD; and (4) study published in English. Exclusion criteria were as follows: (1) non-genetic studies; (2) other than case-control study design; (3) studies focusing on the antidepressant response; (4) in vitro and animal studies; (5) enrolled patients who had depressive episodes due to other psychiatric disorder or studies evaluating other phenotypes in depressive patients (suicide etc.); (6) articles with only an abstract and no full text available; and (7) review articles, meta-analysis, case studies, commentary, and editorials.

#### Acquisition of Antidepressant Response–Associated Studies—

To retrieve genetic association studies related to antidepressant response, the search string using “antidepressants”, “SSRIs”, “MAOIs”, “SNRIs”, “TCAs”, “Atypical antidepressants” AND “pharmacogenetics”, “pharmacogenomics”, genetics”, “gene”, “SNP”, “polymorphism”, “variants” with AND/OR was conducted. Articles were extracted until June 2021. Manual screening of the titles and abstracts and further full-text evaluation was performed. Furthermore, bibliographic cross-reference was also included for additional studies. Articles were included if (1) the patients had a primary diagnosis of major depressive disorder; (2) the study examined the association between the candidate or genome-wide variants and antidepressant response; (3) the study should have dichotomous groups (e.g., between responder and non-responder or remitter and non-remitter); (4) response/ remission assessed by using relevant severity scales such as Hamilton Rating Scale for Depression (HAMD), Montgomery and Åsberg Depression Rating Scale (MADRS) etc.; and (5) the study was published in the English language. Articles were excluded from our study in cases of (1) non-genetic studies; (2) studies other than antidepressant response; (3) studies focusing on disease-associated variants or studies with pharmacokinetic outcomes without gene/variant association; (4) enrolled patients who had depressive episodes due to other psychiatric condition; (5) in vitro and animal studies; (6) articles with only abstract and no full text available; and (7) review articles, meta-analyses, and case studies.

### Data Extraction

Data from selected full-text articles were extracted by P.S. and A.S. and checked by R.K. and S.T. Full-text articles were further segregated into candidate and genome-wide genetic studies. The following data were extracted from eligible articles: (1) study details (author, publication year, PubMed ID); (2) population characteristics (gender, age, ethnicity/country/race); (3) clinical characteristics (phenotype definition, diagnostic criteria, overall sample size); (4) genetic variant details (gene, genetic polymorphism, risk allele, odds ratio [OR], confidence interval, unadjusted *P* value, study minor allele frequency [MAF], genotyping method). Studies in which variant(s) showed statistically significant (*P* < .05) association were considered a positive association. The characteristics of included studies are summarized in supplementary Tables 1–4. We preferred reporting allelic association over genotypic if both were significant. In cases where only genotypic association was reported in the study, we calculated the allelic *P* value using raw data (such as allele frequency or minor allele frequency) and checked for significance and, if found significant, then reported the allelic *P* value.

Next, we checked for reproducible genetic variants and genes that showed significant association in at least 2 studies. The detailed data of reproducible genetic variants and genes in candidate association studies, GWAS studies, and in both (candidate and GWAS) are represented in Tables 1–4.

**Table 1.** List of all Reproducible Genetic Variants With Their Sensitivity, Specificity, and PPV in Candidate Studies, GWAS Studies, and in Both Related to MDD Susceptibility

S. No.	Variant [No. of studies]	Gene	Location/position	Variant type	Polymor­phism (reference/alternate)	Global MAF	Phenotype	Sample size (case/control)	Population/country	Reported risk allele/genotype	OR 95% [CI]	*P*	References	Calculated risk allele	Sensitivity	Specificity	PPV
Candidate variants																	
1	rs774676466; 5HTTLPR [10]	*SLC6A4/5HTT/SERT*	17:30237299	44bp deletion: Upstream gene variant	L/S	0.19 (ALFA)	MDD	35/33	Colombian	S	2.75 [0.876-8.637]	.038	(Perez-Olmos et al., 2016)	S	0.53	0.57	0.55
							MDD	70/142	Spanish	S	2.03	<.05	(Dorado et al., 2007)				
							Depression	184/158	Han Chinese	S	1.817 [1.260–2.619]	.01	(M. Q. Cao et al., 2007)				
							Depression	184/360	German	S	1.4013 [1.086-1.808]^a^	.009	(Grünblatt et al., 2006)				
							MDD	466/836	German	S	1.26 [1.07-1.48]	.0068	(Hoefgen et al., 2005)				
							Major depression	53/107	Racially mixed	LL vs LS vs SS	NA	.008	(Mann et al., 2000)				
							Depression	57/38	Racially mixed	SS [SS vs LS + LL]	5.31 [1.13–25.11]	.02	(Steffens et al., 2002)	S	–	–	–
							MDD	459/412	Han Chinese	LS vs LL + SS	1.42 [1.05-1.91]	.02	(N. Sun et al., 2016)	L	0.36	0.69	0.54
							Depression	366/327	Australia/European	S	0.75 [0.603-0.930]^a^	.0088	(Wray et al., 2009)				
							MDD	401/391	Han Chinese	L	1.446 [1.143–1.830]	.002	(K. Zhang et al., 2009)				
2	rs6265 [8]	*BDNF*	11:27658369	SNV: Missense variant	G/A	0.20 (1000 Genome)	MDD	300/300	Malaysian	AA [AA vs GG + GA]	2.05 [1.48–3.65]	.015	(Aldoghachi et al., 2019)	A	0.40	0.65	0.53
							MDD	45/45	NA	A	NA	.011	(Youssef et al., 2018)				
							Depressive disorder	42/41	Czech population	AA [GG vs GA vs AA]	NA	.0113^a^	(Zeman et al., 2010)				
							Unipolar MD	245/94	White	A [GG vs GA + AA]	1.92 [1.09-3.38]	.024	(Taylor et al., 2007)				
							MDD	110/171	Chinese	A	1.807[1.283-2.546]^a^	.001	(Hwang et al., 2006)				
							Recurrent MDD	116/218	Polish	C	1.72 [1.06–2.79]	.027^a^	(Suchanek et al., 2011)	G	0.78	0.31	0.53
							Major depression	202/346	Chinese	C	1.296 [1.014–1.658]	.039	(R. F. Sun et al., 2011)				
							MDD	272/264	Mexican-American	G	1.66 [1.14-0.41]	.009	(Licinio et al., 2009)				
3	rs4680 [6]	*COMT*	22:19963748	SNV: Missense variant	G/A	0.36 (1000 Genome)	Depression (postmeno­pau­sal women)	332/219	Polish	A [GG vs GA + AA]	2.171 [1.203–3.920]	.009	(Rozycka et al., 2016)	A	0.50	0.49	0.49
							MDD	613/463	Italian	GA vs GG + AA	NA	.03	(Gabriela Nielsen et al., 2015)				
							MDD	368/219	Han Chinese	AG	1.52 [1.04–2.21]	.02	(Shen et al., 2014)				
							Depression	405/2151	Swedish	A [AA + AG vs GG]	1.49 [1.11–2.00]	.009	(Aberg et al., 2011)				
							Depressive disorder	75/135	Japanese	GG vs AG vs AA	2.19 [1.19–4.03]	.0161	(Ohara et al., 1998)				
							EO-MDD	120/628	European	G	1.48[1.09-1.91]	.009	(Massat et al., 2005)	G	–	–	–
4	rs1801133 [4]	*MTHFR*	1:11796321	SNV: Missense variant	C/T	0.24 (1000 Genome)	Depression (perimeno­pausal women)	54/102	Polish	T	1.969 [1.202–3.226]	.007	(Rozycka et al., 2016)	T	0.42	0.68	0.56
							Depression (postmeno­pausal women)	113/219		T	1.812 [1.294–2.538]	.0005					
							MDD	368/219	Han Chinese	T	1.81 [1.40–2.34]	<.001	(Shen et al., 2014)				
							Depressive episode	100/89	Northern Irish	T [TT + TC vs CC]	1.90 [1.00–3.62]	.03	(Kelly et al., 2004)				
							Major depression	32/419	Japanese	TT	2.8 [1.3–6.4]	.005	(Arinami et al., 1997)				
5	rs2242446 [4]	*SLC6A2*	16:55656513	SNV: 5’UTR variant	C/T	0.24 (1000 Genome)	MDD	579/437	Han Chinese	T	1.250 [1.031-1.517]	.023	(Min et al., 2009)	T	0.72	0.34	0.52
							MDD	145/164	Japanese	CC vs TC vs TT	NA	.02	(Inoue et al., 2004)				
							Major depression	388/388	Han Chinese	C	1.33 [1.07–1.65]	.011	(N. Sun et al., 2008)	C	0.40	0.62	0.51
							Major depression	112/136	Korean	C[TT vs. TC + CC]	1.86 [1.11–3.12]	.019	(Ryu et al., 2004)				
6	rs5443 [4]	*GNB3*	12:6845711	SNV: Synonymous variant	C/T	0.49 (1000 Genome)	Major depression	512/513	Han Chinese	C	0.77 [0.65-0.92]	0	(J. Ma et al., 2017)	T	0.55	0.55	0.55
							Depression	184/158	Han Chinese	T	2.214 [1.619–3.029]	.001	(M. Q. Cao et al., 2007)				
							MDD	106/133	Korean	T	1.46 [1.02–2.10]	.041	(H. J. Lee et al., 2004)				
							Major depression	78/111	German	T	1.7952[1.164-2.7689]^a^	.008^a^	(Zill et al., 2004)				
7	rs1045642 [4]	*ABCB1*	7:87509329	SNV: Synonymous variant	T/C	0.39 (1000 Genome)	rDD	90/96	Polish	T	1.661 [1.101 -2.505]^a^	.015^a^	(Jelen et al., 2015)	T	0.43	0.59	0.51
							MDD	631/1100	Japanese	T	1.16 [1.01-1.34]	.034	(Fujii et al., 2012)				
							MDD	54/70	Turkish	C	1.82 [1.095-3.024]^a^	.02	(Ozbey et al., 2014)	C	0.62	0.53	0.57
							MDD	21/42	Portuguese	T	0.36 [0.140–0.920]	.018	(Santos et al., 2014)				
8	rs9340799 [4]	*ESR1*	6:151842246	SNV: Intron variant	A/G	0.28 (1000 Genome)	Depression (postmeno­pausal women)	113/217	Polish	G	0.703 [0.503–0.981]	.037	(Rozycka et al., 2016)	A	0.62	0.38	0.51
							Severe late life current MDD	454/3071	French Caucasian	G [AA vs GG]	0.60 [0.41–0.88]	.009	(Ryan et al., 2011)				
							MDD	89/126	Han Chinese	A	1.6703 [1.0769-2.5908]	.02	(Tsai SJ et al., 2003)				
							MDD	101/95	Turkish	G [GG vs AA + AG]	2.71 [1.10 - 7.29]	.028	(Ozsoy et al, 2021)	G	–	–	–
9	rs6295[3]	*5HTR1A*	5:63962738	SNV: Upstream transcript variant	C/G	0.45 (1000 Genome)	MDD	401/391	Han Chinese	C	0.763 [0.609–0.956]	.018	(K. Zhang et al., 2009)	G	0.33	0.75	0.56
							MDD	400/400	Han Chinese	G	1.4417 [1.153-1.802]^a^	.001	(Wu et al., 2008)				
							MDD	129/134	Ontario (Majority Caucasians)	G	#1.8439 [1.3022-2.6109]	.0006	(Lemonde et al., 2003)				
10	rs2234693 [3]	*ESR1*	6:151842200	SNV: Intron variant	T/C	0.44 (1000 Genome)	Severe late life current MDD	454/3071	French Caucasian	**C** [TT vs CC]	0.61 [0.44–0.84]	.003	(Ryan et al., 2011)	T	0.58	0.46	0.52
							MDD	89/126	Han Chinese	T	1.7642 [1.1911-2.6131]^a^	.004	(Tsai SJ et al., 2003)				
							MDD	101/95	Turkish	CC vs TT + TC	2.05 [1.00-4.34]	.049	(Ozsoy et al, 2021)	C	–	–	–
11	rs4291 [3]	*ACE*	17:63476833	SNV: Upstream transcript variant	T/A	0.34 (1000 Genome)	MDD	187/207	North­eastern Thai	A	0.702 [0.508–0.971]	.04	(Angunsri et al., 2009)	T	0.38	0.68	0.54
							Unipolar depression	642/608	German	TT (TT vs TA vs AA]	NA	.00076	(Baghai et al., 2006)				
								201/245	German Caucasian	TT (TT vs TA vs AA]	NA	.0043					
							Depression	255/750	French	TT	0.44 [0.27–0.71]	.001	(Ancelin et al., 2013)	A	–	–	–
12	rs242939 [3]	*CRHR1*	17:45818213	SNV: Intron variant	C/G	0.11 (1000 Genome)	Recurrent MDD	256/272	Han Chinese	A	0.5271 [0.3491–0.7958]	.0069*	(Z. Liu et al., 2013b)	G	0.13	0.93	0.66
							Recurrent MDD	181/186	Han Chinese	G	2.201 [1.291 -3.75]^a^	.018*	(Xiao et al., 2011)				
							Major depression	206/195	Han Chinese	G	NA	.0008	(Z. Liu et al., 2006)				
13	rs6311 [3]	*5-HTR2A*	13:46897343	SNV: upstream transcript variant	G/A	0.44 (1000 Genome)	MDD	300/300	Han Chinese	T	0.722 [0.574-0.910]^a^	.006	(S. Cao et al., 2015)	G	0.46	0.61	0.54
							MDD	189/148	Korean population	G	1.52 [1.12–2.06]	.007	(Choi et al., 2004)				
							Depressed mood	377/1215	Swedish	AA [AA vs GG]	1.5 [1.05–2.15]	.028	(Jansson et al., 2003)	A	–	–	–
14	rs1360780 [3]	*FKBP5*	6:35639794	SNV: Intron variant	T/C	0.32 (1000 Genome)	MDD	218/742	Polish	TT vs TC vs CC	NA	.011	(Szczepan­kiewicz et al., 2014)	T	0.32	0.72	0.53
							MDD	1256/634	White Non-Hispanic	TT vsTC vs CC	TT vs TC: 0.72 [0.51–1.01]; TT vs TC: 1.01 [0.74–1.40]; TC vs CC:1.394 [1.137-1.709]	.0038	(Lekman et al., 2008)				
							Recurrent unipolar depression	268/284	German	C	1.31 [1.01–1.70]	.0356	(Zobel et al., 2010)	C	–	–	–
15	rs4713916 [3]	*FKBP5*	6:35702206	SNV: Genic upstream transcript variant	A/G	0.22 (1000 Genome)	MDD	218/742	Polish	AA vs AG vs GG	NA	.038	(Szczepan­kiewicz et al., 2014)	A	0.31	0.73	0.53
							MDD	1256/634	White Non-Hispanic	AA vs AG vs GG	AA vs AG: 0.84 [0.59–1.19]; AA vs GG: 1.08 [0.77–1.52]; AG vs GG:1.29 [1.05–1.58]	.046	(Lekman et al., 2008)				
							Recurrent unipolar depression	268/284	German	G	1.38 [1.07–1.79]	.0135	(Zobel et al., 2010)	G	–	–	–
16	rs1800532 [3]	*TPH1*	11:18026269	SNV: Intron variant	C/A	0.32 (1000 Genome)	Depressive disorder	280/230	Polish	AA	2.416 [1.180–4.947]	.016	(Wigner et al., 2018b)	A	0.41	0.65	0.53
							MDD	115/105	Taiwanese	A	1.9703 [1.3285-2.9222]^a^	.000692^a^	(H. C. Wang et al., 2011)				
							MDD	217/395	Finnish/Caucasian	C	1.437 [1.132-1.824]^a^	.003	(Viikki et al., 2010)	C	–	–	–
17	rs1006737 [2]	*CACNA1C*	12:2236129	SNV: Intron variant	G/A	0.30 (1000 Genome)	MDD	1045/1235	Han Chinese	A	1.425 [1.160-1.752]	.0007	(He et al., 2014)	A	0.31	0.78	0.59
							Recurrent major depression	1196/11373	British Isles (European)	A	1.17 (1.07–1.27)	.000711^a^	(Green et al., 2010)				
18	rs4880 [2]	*SOD2*	6:159692840	SNV: Missense variant	T/C	0.41 (1000 Genome)	Depressive disorder	281/229	Polish	TT	2.524 [1.308–6.096]	.008	(Wigner et al., 2018a)	T	0.46	0.61	0.54
							rDD	91/83	Polish	T	1.8 [1.18–2.77]	.006^a^	(Galecki et al., 2010)				
19	rs1801131 [2]	*MTHFR*	1:11794419	SNV: Missense variant	A/C	0.24 (1000 Genome)	MDD	414/257	Italian	CC vs AA + AC	1.97[1.03–3.61]	.01	(Gabriela Nielsen et al., 2015)	C	0.33	0.71	0.53
							MDD	134/143	Slovak	C	1.52 [1.07 – 2.17]	.019	(Evinova et al., 2012)				
20	rs2522833 [2]	*PCLO*	7:82824392	SNV: Missense variant	A/C	0.40 (1000 Genome)	MDD	522/375	Italian	C [CC vs AA + AC]	1.22 [1.07–1.38]	.005	(Minelli et al., 2012)	C	–	–	–
							MDD	238/691	French	C [AA vs CA + CC	0.65 [0.47 0.88]	.0058	(Ryan et al., 2016)	A			
21	rs1800629 [2]	*TNF-α*	6:31575254	SNV: Upstream transcript variant	G/A	0.09 (1000 Genome)	Major depression	50/240	Italian	GG [GG vs Ga vs AA]	2.433 [1.09-5.43]	.007	(Cerri et al., 2009)	G	–	–	–
							MDD	108/125	Korean	A	2.242 [1.219–4.121]	.0125	(Jun et al., 2003)	A			
22	rs4295 [2]	*ACE*	17:63478937	SNV: Genic upstream transcript variant	C/G	0.36 (1000 Genome)	Depression	255/750	French	GG	0.45 [0.28–0.72]	.001	(Ancelin et al., 2013)	C	–	–	–
							Unipolar depression	642/608	German	GG [GG vs GC vs CC]	NA	.0011	(Baghai et al., 2006)	G			
23	rs4343 [2]	*ACE*	17:63488670	SNV: Synonymous variant	G/A	0.35 (1000 Genome)	MDD	191/104	Iranian	G	1.52 [1.07–2.18]	.015	(N. Firouzabadi et al., 2012)	G	–	–	–
							Depression	255/750	French	GG	0.54 [0.35–0.84]	.006	(Ancelin et al., 2013)	A			
24	rs41423247	*NR3C1*	5:143399010	SNV: intron variant	G/C	0.25 (1000 Genome)	rDD	181/149	Polish	C	2.11 [1.53–2.92]	<.00001	(Gałecka et al., 2013)	C	–	–	–
							rDD	419/496	German	GG vs GC + CC	1.8[1.04.–3.2]	.03	(Van Rossum et al., 2006)	G			
GWAS variants																	
1	rs2273289 [2]	*PLOD1*	1:11958233	SNV: Intron variant	T/C	0.25 (1000 Genome)	MDD	1522/1588	European	C	1.414	3.19E-05	(Schosser et al., 2013)	C	–	–	–
							Recurrent depression	1766/1745	European	C	1.377	8.16E-06	(Lewis et al., 2010)				
2	rs2715148 [2]	*PCLO*	7:82820719	SNV: Intron variant	A/C	0.34 (1000 Genome)	MDD	1942/4565	European	A	0.79 [0.73-0.86]	3.89E-08	(Mbarek et al., 2017)	C	–	–	–
							MDD	1738/1802	European	A	0.79 [0.72-0.87]	1.00E-06	(Sullivan et al., 2009)				
3	rs2423618 [2]	*LINC00687*	20:11828794	SNV: Intron variant	T/C	0.31 (1000 Genome)	MDD	1522/1588	European	C	1.312	2.21E-05	(Schosser et al., 2013)	C	–	–	–
							Recurrent depression	1766/1745	European	C	1.262	2.12E-05	(Lewis et al., 2010)				
GWAS and candidate variants																	
1	rs2522833 [3]	*PCLO*	7:82824392	SNV: Missense variant	A/C	0.40 (1000 Genome)	MDD	1738/1802	European	C	1.26 [1.15-1.39]	2.00E-06	(Sullivan et al., 2009)	C	–	–	–
							MDD	522/375	Italian	C [CC vs AA + AC]	1.22 [1.07–1.38]	.005	(Minelli et al., 2012)				
							MDD	238/691	French	C [AA vs CA + CC	0.65 [0.47 0.88]	.0058	(Ryan et al., 2016)	A			
2	rs2715147 [2]	*PCLO*	7:82819089	SNV: Intron variant	C/A	0.35 (1000 Genome)	MDD	1738/1802	Western European	C	0.79	1.50E-06	(Verbeek et al., 2013)	A	–	–	–
							MDD	1942/4565	European	C	0.79 [0.73-0.86]	3.89E-08	(Mbarek et al., 2017)				
3	rs9416742 [2]	*BICC1*	10:58782934	SNV: Intron variant	G/A	0.07 (1000 Genome)	Recurrent depression	1766/1745	European	A	0.719	1.30E-07	(Lewis et al., 2010)	G	–	–	–
							MDD	62/306	French	A [GG vs GA + AA]	0.48 [0.24 0.96]	.038	(Ryan et al., 2016)				
4	rs999845 [2]	*BICC1*	10:58776160	SNV: Intron variant	T/C	0.17 (1000 Genome)	Recurrent depression	1766/1745	European	A	0.7273	3.12E-07	(Lewis et al., 2010)	C	–	–	–
							MDD	62/306	French	A [GG vs GA + AA]	0.49 [0.25 0.94]	.033	(Ryan et al., 2016)				

Abbreviations: MAF, minor allele frequency; OR, odds ratio; CI, confidence interval; P, p value; PPV, positive predictive value; SNV, single nucleotide variant; bp, base pair, ALFA, allele frequency aggregator; MDD, Major depressive disorder; L/S, long/short; EO-MDD, early onset- major depressive disorder; 5'UTR, 5' untranslated region; rDD, recurrent depressive disorder; NA, not available

^a^Self calculated values.

**Table 2.** List of All Reproducible Genes in GWAS Studies, Candidate Studies, and in Both Related to MDD Susceptibility

S. No.	Gene	Name	Location	Phenotype	Sample size (case/control)	Population/country	Reference	Function
Candidate genes								
1	*SLC6A4/5HTT/SERT*	Solute carrier family 6 member 4	17q11.2	MDD	74/150	Han Chinese	(Ran et al., 2020)	Monoamine transport
				MDD	35/33	Colombian	(Perez-Olmos et al., 2016)	
				MDD	459/412	Han Chinese	(N. Sun et al., 2016)	
				Depression	366/327	Australia/European	(Wray et al., 2009)	
				MDD	401/391	Han Chinese	(K. Zhang et al., 2009)	
				MDD	272/264	Mexican Americans	(Dong et al., 2009)	
				MDD	70/142	Spanish	(Dorado et al., 2007)	
				Depression	184/158	Han Chinese	(M. Q. Cao et al., 2007)	
				Depression	184/360	German	(Grünblatt et al., 2006)	
				MDD	466/836	German	(Hoefgen et al., 2005)	
				Depression	57/38	Racially mixed	(Steffens et al., 2002)	
				Major depression	53/107	Racially mixed	(Mann et al., 2000)	
				Unipolar depression	33/362	Han Chinese	(W. Liu et al., 1999)	
				Major depression with melancholia	74/84	Spanish	(Gutiérrez et al., 1998)	
				Unipolar depressive disorder	39/193	Edinburgh (UK)	(Ogilvie et al., 1996)	
2	*BDNF*	Brain derived neurotrophic factor	11p14.1	MDD	300/300	Malaysian	(Aldoghachi et al., 2019)	Promotes neuronal survival in the adult brain
				MDD	45/45	NA	(Youssef et al., 2018)	
				Recurrent MDD	116/218	Polish	(Suchanek et al., 2011)	
				Major depression	202/346	Chinese	(R. F. Sun et al., 2011)	
				MDD	272/264	Mexican American	(Licinio et al., 2009)	
				Depressive disorder	42/41	Czech	(Zeman et al., 2010)	
				Unipolar MD	245/94	White	(Taylor et al., 2007)	
				MDD	110/171	Chinese	(Hwang et al., 2006)	
				MDD	456/1097	German	(Schumacher et al., 2005)	
3	*TPH2*	Tryptophan hydroxylase 2	12q21.1	Depressive disorder	280/230	Polish	(Wigner et al., 2018b)	Biosynthesis of serotonin
				Major depression	289/289	Han Chinese	(J. Ma et al., 2015)	
				MDD	90/182	Han Chinese	(Shen et al., 2011)	
				Major depression	117/83	Chinese	(Y. M. Lin et al., 2009b)	
				MDD	508/463	Han Chinese	(Tsai et al., 2009b)	
				Unipolar major depression	87/219	Majority Caucasians	(X. Zhang et al., 2005)	
				Major depression	300/265	German	(Zill et al., 2004)	
4	*ABCB1*	ATP binding cassette subfamily B member 1	7q21.12	rDD	90/96	Polish	(Jelen et al., 2015)	Transportation of various molecules across extra- and intra-cellular membranes; involved in multidrug resistance
				MDD	54/70	Turkish	(Ozbey et al., 2014)	
				MDD	21/42	Portuguese	(Santos et al., 2014)	
				MDD	631/1100	Japanese	(Fujii et al., 2012)	
				MDD	272/264	Mexican Americans	(Dong et al., 2009)	
				Depression	284/331	Mexican Americans	(Wong et al., 2008)	
5	*MTHFR*	Methylenetetrahydrofolate reductase	1p36.22	Depression (perimenopausal women)	54/102	Polish Caucasian	(Rozycka et al., 2016)	A co-substrate for homocysteine remethylation to methionine; neural tube defects, folate-sensitive
				Depression (postmenopausal women)	113/219			
				MDD	414/257	Italian	(Gabriela Nielsen et al., 2015)	
				MDD	368/219	Han Chinese	(Shen et al., 2014)	
				MDD	134/143	Slovak	(Evinova et al., 2012)	
				Depressive episode	100/89	Northern Irish	(Kelly et al., 2004)	
				Major depression	32/419	Japanese	(Arinami et al., 1997)	
6	*COMT*	Catechol-O-methyltransferase	22q11.21	Depression (postmenopausal women)	332/219	Polish Caucasian	(Rozycka et al., 2016)	Catalyzes the transfer of a methyl group from S-adenosylmethionine to catecholamines (dopamine, epinephrine, and norepinephrine)
				MDD	613/463	Italian (Caucasian)	(Gabriela Nielsen et al., 2015)	
				MDD	368/219	Han Chinese	(Shen et al., 2014)	
				Depression	405/2151	Swedish	(Aberg et al., 2011)	
				Depressive disorder	75/135	Japanese	(Ohara et al., 1998)	
				EO-MDD	120/628	European	(Massat et al., 2005)	
7	*ACE*	Angiotensin I converting enzyme	17q23.3	Depression	255/750	French	(Ancelin et al., 2013)	Blood pressure regulation and electrolyte balance
				MDD	191/104	Iranian	(N. Firouzabadi et al., 2012)	
				MDD	187/207	Northeastern Thai	(Angunsri et al., 2009)	
				Unipolar depression	642/608	German (majority) Caucasian	(Baghai et al., 2006)	
8	*CRHR1*	Corticotropin releasing hormone receptor 1	17q21.31	Recurrent MDD	256/272	Han Chinese	(Z. Liu et al., 2013b)	G protein-coupled receptor activity; major regulator of the hypothalamic-pituitary-adrenal pathway
				MDD	173/285	Japanese	(Ishitobi et al., 2012)	
				Recurrent MDD	181/186	Han Chinese	(Xiao et al., 2011)	
				Major depression	206/195	Han Chinese	(Z. Liu et al., 2006)	
9	*GNB3*	G protein subunit beta 3	12p13.31	Major depression	512/513	Han Chinese	(J. Ma et al., 2017)	Integrate signals between receptors and effector proteins
				Depression	184/158	Han Chinese	(M. Q. Cao et al., 2007)	
				MDD	106/133	Korean	(H. J. Lee et al., 2004)	
				Major depression	78/111	German	(Zill et al., 2004)	
10	*SLC6A2/NET*	Solute carrier family 6 member 2	16q12.2	MDD	579/437	Han Chinese	(Min et al., 2009)	Regulates norepinephrine homeostasis
				Unipolar MD	426/643	German	(Haenisch et al., 2009)	
				Major depression	388/388	Han Chinese	(N. Sun et al., 2008)	
				Major depression	112/136	Korean	(Ryu et al., 2004)	
				MDD	145/164	Japanese	(Inoue et al., 2004)	
11	*NR3C1*	Nuclear receptor subfamily 3 group C member 1	5q31.3	MDD	251/307	German Caucasian	(Sarubin et al., 2017)	Involved in inflammatory responses, cellular proliferation, and differentiation in target tissues
				rDD	181/149	Polish	(Gałecka et al., 2013)	
				MDD	193/732	Polish	(Szczepankiewicz et al., 2011)	
				Depression	284/331	Mexican Americans	(Wong et al., 2008)	
				MDD	180/173	Belgian	(van West et al., 2006)	
12	*TPH1*	Tryptophan hydroxylase 1	11p15.1	Depressive disorder	280/230	Polish	(Wigner et al., 2018b)	Biosynthesis of serotonin
				MDD	115/105	Taiwanese	(H. C. Wang et al., 2011)	
				MDD	217/395	Finnish/Caucasian	(Viikki et al., 2010)	
				Depression	30/86	Taiwanese	(H. S. Sun et al., 2004)	
13	*CACNA1C*	Calcium voltage-gated channel subunit alpha1 C	12p13.33	MDD	1045/1235	Han Chinese	(He et al., 2014)	Calcium channels; mediate the influx of calcium ions upon membrane polarization.
				MDD	640/542	German	(Kloiber et al., 2012)	
				Recurrent major depression	1196/11373	British Isles (European)	(Green et al., 2010)	
14	*FKBP5*	FKBP prolyl isomerase 5	6p21.31	MDD	218/742	Polish	(Szczepankiewicz et al., 2014)	Role in immunoregulation; involving protein folding and trafficking
				MDD	1256/634	White Non-Hispanic	(Lekman et al., 2008)	
				Recurrent unipolar depression	268/284	German (Caucasian)	(Zobel et al., 2010)	
15	*PCLO*	Piccolo presynaptic cytomatrix protein	7q21.11	MDD	238/691	French	(Ryan et al., 2016)	Synaptic vesicle trafficking
				MDD	1738/1802	Western European	(Verbeek et al., 2013)	
				MDD	522/375	Italian	(Minelli et al., 2012)	
16	*5-HTR2A*	5-hydroxytryptamine receptor 2A	13q14.2	MDD	300/300	Han Chinese	(S. Cao et al., 2015)	Serotonergic neurotransmission
				MDD	189/148	Korean (Caucasian)	(Choi et al., 2004)	
				Depressed mood	377/1215	Swedish (Caucasians)	(Jansson et al., 2003)	
17	*CNR1*	Cannabinoid receptor 1	6q15	Major depression (Melancholic)	151/150	Spanish	(Mitjans et al., 2013)	G protein-coupled receptor activity and cannabinoid receptor activity
				Recurrent MD	83/117	Italian (Caucasian)	(Monteleone et al., 2010)	
18	*CRY1*	Cryptochrome circadian regulator 1	12q23.3	MDD	383/4154	Finnish	(Kovanen et al., 2017)	Regulates the circadian clock
				Dysthymia	166/4154			
				Depressive disorder	479/4154			
				MDD	105/485	Chinese	(Hua et al., 2014)	
19	*DRD2*	Dopamine receptor D2	11q23.2	MDD	403/475	Han Chinese	(Y. Wang et al., 2012)	Dopamine receptor; include G protein-coupled receptor activity
				MDD	177/160	Estonian	(Koks et al., 2006)	
20	*GSK3B*	Glycogen synthase kinase 3 beta	3q13.33	MDD	1045/1235	Han Chinese	(Chen et al., 2015)	A negative regulator of glucose homeostasis; involved in energy metabolism, inflammation, ER-stress, mitochondrial dysfunction, and apoptotic pathways
				MDD	447/432	Han Chinese	(K. Zhang et al., 2010)	
21	*HTR1A*	5-hydroxytryptamine receptor 1A	5q12.3	MDD	331/804	Japanese	(Kishi et al., 2009b)	Serotonergic neurotransmission; G protein-coupled serotonin receptor activity
				Unipolar MD	426/643	German	(Haenisch et al., 2009)	
				MDD	401/391	Han Chinese	(K. Zhang et al., 2009)	
				MDD	400/400	Northern Han Chinese	(Wu et al., 2008)	
				MDD	129/134	Ontario (Majority Caucasians)	(Lemonde et al., 2003)	
22	*KCNK2*	Potassium 2 pore domain channel subfamily K member 2	1q41	MDD	590/441	Italian (Caucasian)	(Congiu et al., 2015)	Potassium channel activity
				MDD	449/421	Han Chinese	(Liou et al., 2009)	
23	*TNF-α*	Tumor necrosis factor	6p21.33	Major depression	50/240	Italian (Caucasian)	(Cerri et al., 2009)	Multifunctional proinflammatory cytokine; involved in cell proliferation, differentiation, apoptosis, lipid metabolism, and coagulation
				MDD	108/125	Korean	(Jun et al., 2003)	
24	*ESR1/ER-α*	Estrogen receptor 1	6q25.1-q25.2	MDD	125/120	Turkish	(Ozsoy et al, 2021)	DNA-binding transcription factor activity and enzyme binding
				Depression (postmenopausal women)	113/219	Polish Caucasian	(Rozycka et al., 2016)	
				Severe late life current MDD	454/3071	French	(Ryan et al., 2011)	
				MDD	89/126	Han Chinese	(Tsai et al., 2003)	
25	*ESR2/ ER-β*	Estrogen receptor 2	14q23.2-q23.3	Severe late life current MDD	454/3071	French	(Ryan et al., 2011)	DNA-binding transcription factor activity and enzyme binding
				MDD	102/150	Han Chinese	(Y. G. Geng et al., 2007)	
26	*sod2*	Superoxide dismutase 2	6q25.3	Depressive disorder	281/229	Polish	(Wigner et al., 2018a)	A mitochondrial protein binds to the superoxide byproducts and converts them to hydrogen peroxide and diatomic oxygen
				rDD	91/83	Polish	(Galecki et al., 2010)	
27	*MAOA*	Monoamine oxidase A	Xp11.3	Depression (perimenopausal women)	54/102	Polish Caucasian	(Rozycka et al., 2016)	Monoamine transport; oxidoreductase activity
				Depression (postmenopausal women)	113/219			
				MDD	228/213	Chinese	(Yu et al., 2005)	
				Recurrent MDD	73/68	German	(Schulze et al., 2000)	
GWAS genes								
1	*ARHGAP8*	Rho GTPase activating protein 8	22q13.31	MDD	203/196	Mexican American and European	(Wong et al., 2017)	Regulates cell processes
				Recurrent MDD	926/866	European	(Muglia et al., 2010)	
2	*CSMD1*	CUB and Sushi multiple domains 1	8p23.2	Major depression	604/1364	German	(Rietschel et al., 2010)	–
				Recurrent early-onset MDD	724/720	European	(Shi et al., 2011)	
3	*FHIT*	Fragile histidine triad diadenosine triphosphatase	3p14.2	Recurrent early-onset MDD	296/916	European	(Shi et al., 2011)	Involved in purine metabolism
				Recurrent MDD	926/866	European	(Muglia et al., 2010)	
4	*LHPP*	Phospholysine phosphohistidine inorganic pyrophosphate phosphatase	10q26.13	Recurrent MDD	5303/5337	Han Chinese	(Consortium, 2015)	Metabolism of nucleotides
				Recurrent MDD	926/866	European	(Muglia et al., 2010)	
5	*PCLO*	Piccolo presynaptic cytomatrix protein	7q21.11	MDD	1942/4565	European	(Mbarek et al., 2017)	Synaptic vesicle trafficking
				MDD	1738/1802	European	(Sullivan et al., 2009)	
6	*PLOD1*	Procollagen-lysine,2-oxoglutarate 5-dioxygenase 1	1p36.22	MDD	1522/1588	European	(Schosser et al., 2013)	Protein homodimerization activity and iron ion binding
				Recurrent depression	1766/1745	European	(Lewis et al., 2010)	
7	*LINC00687*	Long intergenic non-protein coding RNA 687	20p12.2	MDD	1522/1588	European	(Schosser et al., 2013)	–
				Recurrent depression	1766/1745	European	(Lewis et al., 2010)	
8	*LOC100996549*	Uncharacterized LOC100996549	2p25.1	MDD	1522/1588	European	(Schosser et al., 2013)	–
				Recurrent MDD	492/1052	European	(Muglia et al., 2010)	
Candidate and GWAS genes								
1	*PCLO*	Piccolo presynaptic cytomatrix protein	7q21.11	MDD	1942/4565	European	(Mbarek et al., 2017)	Synaptic vesicle trafficking
				MDD	1738/1802	European	(Sullivan et al., 2009)	
				MDD	238/691	French	(Ryan et al., 2016)	
				MDD	1738/1802	Western European	(Verbeek et al., 2013)	
				MDD	522/375	Italian	(Minelli et al., 2012)	
2	*ESR1*	Estrogen receptor 1	6q25.1-q25.2	Major depression	604/1364	German	(Rietschel et al., 2010)	DNA-binding transcription factor activity; role in growth, metabolism, sexual development
				MDD	125/120	Turkish	(Ozsoy et al, 2021)	
				Depression (postmenopausal women)	113/219	Polish Caucasian	(Rozycka et al., 2016)	
				Severe late life current MDD	454/3071	French	(Ryan et al., 2011)	
				MDD	89/126	Han Chinese	(Tsai et al., 2003)	
3	*APC*	APC regulator of WNT signaling pathway	5q22.2	Recurrent MDD	926/866	European	(Muglia et al., 2010)	Antagonist of the Wnt signaling pathway; cell migration and adhesion, transcriptional activation, and apoptosis
				MDD	397/473	Han Chinese	(Yang et al., 2012)	
4	*GRM7*	Glutamate metabotropic receptor 7	3p26.1	Recurrent MDD	926/866	European	(Muglia et al., 2010)	Glutamatergic neurotransmission
				MDD	1738/1802	Western European	(Verbeek et al., 2013)	
5	*CNTNAP2*	Contactin associated protein 2	7q35-q36.1	Recurrent MDD	492/1052	European	(Muglia et al., 2010)	Cell adhesion molecules and receptors; mediates interactions between neurons and glia
				MDD	1135/1135	Han Chinese	(W. Ji et al., 2013)	
6	*EHD3*	EH domain containing 3	2p23.1	Recurrent MDD	1418/1918	European	(Muglia et al., 2010)	Role in endocytic transport, Golgi maintenance and morphology
				MDD	283/248	Han Chinese	(L. Wang et al., 2014a)	
7	*BICC1*	BicC family RNA binding protein 1	10q21.1	Recurrent depression	1766/1745	European	(Lewis et al., 2010)	Regulating gene expression by modulating protein translation
				MDD	62/306	French	(Ryan et al., 2016)	

Abbreviations: MDD, major depressive disorder; rDD, recurrent depressive disorder, MD, major depression; EO-MDD, early onset major depressive disorder.

**Table 3.** List of All Reproducible Genetic Variants With Their Sensitivity, Specificity, and PPV in Candidate Studies, GWAS Studies and in Both Related to Antidepressant Response

S.No.	Variant [no. of studies]	Gene	Location:position (GRCh38)	Variant type	Polymor­phism (reference/alternate)	Global MAF	Response status	Drug class	Associated phenotype	Sample size (res­ponder/nonres­ponder)	Population	Reported risk allele/genotype	OR 95% [CI]	*P*	References	Calcu­lated Risk Allele	Sensi­tivity	Speci­ficity	PPV
Candidate variants SNPs																			
1	rs774676466; 5-HTTLPR [22]	*SLC6A4/5HTT/SERT*	17:30237299	Insertion/deletion (44bp)	L/S	0.19 (ALFA)	Response: ≥50% reduction in HAMD, MADRS, Beck score after treatment	SSRIs	Non-responder	148 (65/83)	South Indian	S	7.41 [3.87-14.20]^a^	<.0001^a^	(Mandal et al., 2020)	S	0.70	0.44	0.53
									Non-responder	102 (56/46)	South Indian	S carrier [LL vs LS + SS]	4 [1.45–11.03]	.0066	(Manoharan et al., 2016)				
									Non-responder	104 (65/39)	Iranian	SS [SS vs LS + LL]	2.625 [1.127-6.1139]^a^	.023^a^	(Sahraian et al., 2013)				
									Non-responder	362 (243/119)	Han Chinese	S carrier [LL vs LS + SS]	2.85[1.0687-7.6002]^a^	.03	(Min et al., 2009)				
									Non-responder	130 (65/65)	Croatian	S	2.55 [1.53-4.25]^a^	.0004	(Bozina et al., 2008)				
									Non-reponder	224 (81/143)	Han Chinese/ Taiwanese	S	2.0404 [1.3365-3.1151]^a^	.0009^a^	(Hong et al., 2006)				
									Non-responder	121 (40/81)	Chinese	S	2.0271 [1.1181-3.6754]^a^	.019	(Yu et al., 2002)				
									Non-reponder	80 (68/12)	Japanese	SS[SS vs LS + LL]	8.6842 [1.0609-71.0854]^a^	.043	(Kato et al., 2006)	S	Raw data not available		
									Non- responder	115 (59/56)	Korean	LL [LLvs SS + SL]	6.24 [1.24–31.26]	.026	(Won et al., 2012)	L	0.47	0.61	0.49
									Non- responder	99 (46/42)	Korean	L	3.9011 [2.039-7.4636]	5.2E-05	(Myung et al., 2013)				
									Non-responder	59 (37/22)	Japanese	L	2.6727 [1.085-6.5836]^a^	.037	(Umene-Nakano et al., 2010)				
									Non-responder	119 (69/50)	Koreans	L	3 [1.6135-5.5778]^a^	.0004^a^	(H. Kim et al., 2006)				
									Non-responder	54 (35/19)	Japanese	L	4.342 [1.7524-10.7582]^a^	.001	(Yoshida et al., 2002)				
									Non-responder	120 (87/33)	Korean	L	2.2025 [1.1731-4.135]^a^	.012^a^	(D. K. Kim et al., 2000)				
									Non-responder	239 (154/85)	Korean	ss vs sl + ll	2.18 (1.27–3.75)	.00601	(Lim et al., 2014)	L	Raw data not available		
								SNRIs	Non-responder	179 (80/93)	Majority Caucasi­ans	L	2.17[4.69- 1.002] ^a^	.049	(Calabro et al., 2018)	L	–	–	–
									Non- responders	101 (54/27)	Korean	SS[SS vs LS + LL]	4.40[1.45–13.32]	.006	(S. H. Lee et al., 2010)	S	–	–	–
								TCA	Non-responder	89 (55/34)	Koreans	L	3.3462 [1.6911-6.6211]^a^	.0004^a^	(H. Kim et al., 2006)	L	–	–	–
								Atypical	Non-responder	101 (62/39)	Korean	L	2.8629 [1.2611-6.4993]^a^	.009^a^	(R. H. Kang et al., 2007)	L	–	–	–
								Mixed	TRD	128 (98/30)	Caucasian	L carrier [LL + LS vs SS]	5.0556 [1.1246-22.7263]^a^	.023^a^	(Wilkie et al., 2009)	L	–	–	–
							Remission: MADRS score ≤7 after treatment	SSRIs	Non-remitter	85 (29/56)	Finnish/Caucasian	S carrier [LL vs LS + SS]	3.2143 [1.2471-8.2844]^a^	.01375^a^	(Illi et al., 2011)	S	0.36	0.81	0.57
									Non-remitter	131 (91/40)	Spanish	SS[SS vs LS + LL]	3.23 [1.24–8.5]	.006	(Arias et al., 2003)				
									Non-remitter	27 (16/11)	Caucasi­ans	S carrier [LL vs LS + SS]	10 [1.0256-97.5046]^a^	.04	(Alexopoulos et al., 2009)	S	Raw data not available		
									Non-remitter	362 (156/206)	Han Chinese	LL vs LS vs SS	NA	.007	(Min et al., 2009)	L	–	–	–
2	rs57098334; STin2-VNTR [5]	*SLC6A4*	17:30221568	Insertion/deletion	L/S	0.025-0.15^b^	Response: ≥50% reduction in HAMD score after treatment	SSRIs	Non-responder	119 (69/50)	Koreans	S	18.0759 [4.1284-79.1441]^a^	<.0001^a^	(H. Kim et al., 2006)	S	0.18	0.94	0.63
									Non-responder	362 (239/123)	Han Chinese	10	1.7314 [1.0525-2.8484]	.029	(Min et al., 2009)				
									Non-responder	120 (87/33)	Korean	S	21.25 [6.9629-64.8527]^a^	<.0001^a^	(D. K. Kim et al., 2000)				
									Non-responder	239 (154/85)	Korean	ll vs sl + ss	3.86 (1.90–7.84)	.0002	(Lim et al., 2014)	S	Raw data not available		
								Atypical	Non-responder	283 (100/183)	Korean	10 repeat [10.10 + 12.10 vs 12.12]	2.4375 [1.1595-5.1241]^a^	.016^a^	(Chang et al., 2014)	S	–	–	–
							Remission:HAMD score ≤8 after treatment	SSRIs	Non-remitter	362 (151/211)	Han Chinese	10 carrier [12/12 vs 12/10 + 10/10]	1.7756 [1.000-3.1535]	.048	(Min et al., 2009)	S	–	–	–
3	rs6265 [5]	*BDNF*	11:27658369	SNV: Missense Variant	G/A	0.20 (1000 Gen­o­me)	Response: ≥50% reduction in HAMD score after treatment	SSRIs	Non-responder	298 (219/79)	Chinese	G	2.076 [1.43-3.008]	<.0001^a^	(X. C. Wang et al., 2014b)	G	0.62	0.57	0.36
									Non-responder	83 (57/26)	Korean		2.5 [1.249–5.005]	.009	(Choi et al., 2006)				
									Non-responder	187 (108/52)	Caucasian	GG [GG vs AA + GA]	2.377 [1.170-4.828]	.015	(El-Hage et al., 2015)	G	Raw data not available		
								Mixed	Non-responder	188 (71/117)	European	G	2.0704 [1.22–3.506]	.00629	(Kocabas et al., 2011)	G	–	–	–
								SNRI	Non- responders	117 (23/39)	Han Chinese (Taiwa­nese)	AA	NA	.006	(Chi et al., 2010)	A	–	–	–
4	rs7997012 [4]	*HTR2A*	13:46837850	SNV: Intron Variant	A/G	0.27 (1000 Gen­ome)	Response: ≥50% reduction in QIDS score after treatment	SSRI	Non-responder	935 (675/260)	Mixed (White, Black and other)	G	NA	1.3E-05	(Paddock et al., 2007)	G	–	–	–
							Resistant: Failed to reach an HAMD-17 score < 17, after at least 2 adequate antidep­ressant treatments within the current episode	Mixed	Non-responder (resistant)	206 (58/74)	European	A	1.7402 [1.0309-2.9373]^a^	.037	(Noro et al., 2010)	A	–	–	–
							Remission: HAMD score ≤8/ QIDS-C ≤5 after treatment	SSRI	Non remitter	46 (32/14)	Caucasian	A	5.4[2.0718-14.0746]^a^	.000329^a^	(Gasso et al., 2018)	A	–	–	–
									Non-remitter	NA	Mixed (White, Black and other)	G	NA	9.5E-05	(Paddock et al., 2007)	G	–	–	–
								Mixed	Non-remitter	186 (61/125)	Cauca­sian/Northern Germany	A	1.79­53[1.1521-2.7975]^a^	.00938	(Lucae et al., 2010)	A	–	–	–
5	rs6295 [4]	*5HTR1A*	5:63962738	SNV: Upstream gene variant	C/G	0.45 (1000 Gen­ome)	Response: ≥50% reduction in HAMD score after treatment	SSRIs	Non- responder	222 (83/139)	Han Chinese/ Taiwanese	G	2.0516 [1.2936-3.2536]^a^	.002^a^	(Yu et al., 2006)	G	same cohort		
									Non responder	224 (81/143)	Han Chinese/ Taiwanese	G	2.0598 [1.2893-3.2908]^a^	.002^a^	(Hong et al., 2006)	G			
								Mixed	Non-responders	137 (22/115)	Japanese	C carrier (CC + CG vs GG]	8.2963 [1.713-40.1795]^a^	.0129^a^	(Kato et al., 2009)	C	–	–	–
							Resistant: Failed to reach an HAMD-17 score < 17, after at least 2 adequate antidep­ressant treatments within the current episode	Mixed	Non-responder (resistant)	206 (58/75)	European	C	1.693[1.04–2.77]	.035	(Noro et al., 2010)	C	–	–	–
							Remission: HAMD score ≤7 after 2 weeks of treatment	Mixed	Non-remitters	137 (15/122)	Japanese	G carrier [CC vs CG + GG]	7.375 [1.474-36.913]^a^	.028^a^	(Kato et al., 2009)	G	–	–	–
6	rs5443 [3]	*GNB3*	12:6845711	SNV: Synony­mous Variant	C/T	0.49 (1000 Gen­ome)	Response: ≥50% reduction in HAMD score after treatment	Mixed	Non- responder	163 (58/105)	Caucasian	CC vs CT vs TT	NA	.04	(Wilkie et al., 2007)	C	0.71	0.14	0.66
									Non-responder	106 (67/39)	Korean	CC [CC vs TT + TC]	2.8973 [1.0492-8.0008]^a^	.035	(H. J. Lee et al., 2004)				
									Non- responder	101 (35/66)	Han Chinese/Taiwanese	TT (CC + CT vs TT)	2.8889 [1.0954-7.619]^a^	.029	(E. Lin et al., 2009a)	T	–	–	–
							Remission: HAMD score ≤7 after treatment	Mixed	Non-remitter	110 (70/40)	Caucasian	CC vs CT vs TT	NA	.02	(Wilkie et al., 2007)	T	–	–	–
7	rs334558 [2]	*GSK3B*	3:120094435	SNV:5 prime UTR Variant	T/C	0.40 (1000 Gen­ome)	Response: ≥50% reduction in HAM-D score after treatment	Mixed (SSRIs and SNRI)	Non-responder	143 (90/53)	Japanese	TT vs CC + CT	10.4 [1.332-81.186]^a^	.007^a^	(Sunada et al., 2019)	T	–	–	–
								SSRIs	Non-responder	168 (75/93)	Han Chinese/Taiwanese	T	1.6971 [1.0835-2.6583]^a^	.02	(Tsai et al., 2009b)	T	–	–	–
							Remission: HAM-D score ≤ 7 points after treatment	Mixed	Non-remitter	143 (18/125)	Japanese	TT vs CC + CT	3.986 [1.1972-13.2713]^a^	.032^a^	(Sunada et al., 2019)	T	–	–	–
8	rs2470890 [2]	*CYP1A2*	15:74755085	SNV: Synon­ymous Variant	T/C	0.40 (ALFA)	Remission:HAMD score ≤ 7 points after treatment	SNRI	Non-remitter	175 (97/78)	Han Chinese	T	2.438 [1.234–4.815]	.0087	(Zhu et al., 2019)	T	–	–	–
								SSRI	Non-remitter	171 (69/102)	Han Chinese	C	2.7623 [1.3662-5.585]^a^	.0035^a^	(K. M. Lin et al., 2010)	C	–	–	–
9	rs6313 [2]	*HTR2A*	13:46895805	SNV: Synony­mous Variant	C/T	0.44 (1000 Geno­­me)	Remission:Disease free state for at least 2 weeks	SSRI	Non-remitter	46 (32/14)	Caucasian	T	NA	.0225	(Gasso et al., 2018)	T	–	–	–
					G/C		Remission:MADRS remission (score ≤10)	SSRI	Non-remitter	166	Han Chinese	G	1.69 [1.00-2.84]	.049	(Su et al., 2016)	G	–	–	–
10	rs2066713 [2]	*SLC6A4*	17:30224647	SNV: Intron Variant	C/T	0.25 (1000 Geno­m­e­)­	Response: ≥50% reduction in HAMD score after treatment	SSRI	Non-responder	239 (154/85)	Korean	T	4.0548 [1.9722-8.3366]^a^	.0000802^a^	(Lim et al., 2014)	T	–	–	–
							Remitter: Disease free state for at least 2 weeks	SSRI	Non-remitter	46 (32/14)	Caucasian	T	NA	.038	(Gasso et al., 2018)	T	Raw data not available		
11	rs41423247 [2]	*NR3C1*	5:143399010	SNV: Intron Variant	G/C	0.25 (1000 Gen­ome)	Response:≥50% reduction in HAM-D score after treatment	SSRI	Non- responder	100 (70/30)	Caucasian (Iranian)	C	2.2[1.09–4.44]	.032	(Nouraei et al., 2018)	C	0.55	0.34	0.42
										160 (104/56)	Japanese	C	5.11 [1.14-22.82]^a^	.019	(Takahashi et al., 2014)				
12	rs2171363 [2]	*TPH2*	12:71966484	SNV: Intron Variant	A/G	0.40 (1000 Gen­ome)	Response: ≥50% reduction in HDRS score after treatment	Mixed (SSRIs & SNRIs)	Non- responder	281 (205/76)	Han Chinese	A	1.544 [1.055–2.258]	.02478	(Xu et al., 2016)	A	–	–	–
								SSRIs	Non-responder	187 (126/61)	Han Chinese/Taiwanese	CC vs CT vs TT	NA	.009	(Tsai et al., 2009b)	C	–	–	–
13	rs2075507 [2]	*COMT*	22:19940569	SNV: Upstream Variant	G/A	0.34 (1000 Gen­ome)	Remission:HAMD score ≤ 8 points after treatment	SSRI	Non-remitters	123 (24/35)	Japanese	C	7.5 [2.11-26.63]^a^	.0006^a^	(Fukui et al., 2014)	G	–	–	–
							Resistant: Failed to reach an HAMD-17 score < 17,after at least 2 adequate antide­pressant treatments within the current episode	Mixed	Non-responder (resistant)	166 (61/105)	Caucasian	GG vs GA vs AA	NA	.005	(Kocabas et al., 2010)	G	–	–	–
14	rs1954787 [2]	*GRIK4*	11:120792654	SNV: Intron Variant	A/G	0.49 (1000 Gen­ome)	Response: ≥50% reduction in HDRS/QIDS score after treatment	Mixed (SSRI, SNRI)	Non- responder	281 (205/76)	Han Chinese	A	1.868 [1.173–2.975]	.0078	(Pu et al., 2013)	A	–	–	–
					T/C			SSRI	Non- responder	935 (675/260)	Mixed (White, Black and other)	T	NA	.00023	(Paddock et al., 2007)	T	–	–	–
							Remission:QIDS-C-16 score ≤5	SSRI	Non- remitter	NA	Mixed (White, Black and other)	T	NA	.00034	(Paddock et al., 2007)	T	–	–	–
15	rs1360780 [2]	*FKBP5*	6:35639794	SNV: Intron Variant	T/C	0.32 (1000 Gen­ome)	Early response:≥25% reduction in HAMD score after treatment	Mixed	Non-responder	233 (147/86)	German/Caucasian	C	1.6574 [ 1.072-2.5624]^a^	.02^a^	(Binder et al., 2004)	C	–	–	–
										85 (43/42)			2.3974 [1.2181-4.7186]^a^	.01^a^		C	–	–	–
							Remission:QIDS-C-16 score ≤5 after treatment	SSRIs	Non-remission	799 (516/283)	White non-Hispanic	CC [CC vs TT + CT]	1.43 [1.0685-1.9138]^a^	.015^a^	(Lekman et al., 2008)	C	–	–	–
GWAS Variants																			
1	rs6046805 [2]	*CFAP61*	20:20343697	SNV: Intron Variant	G/A, G/C	0.41 (1000 gen­ome)	Remission	SSRI	Remitter	840/837	Mixed	NA	NA	.01744	(Cocchi et al., 2016)	–	–	–	–
										743/608	Mixed	NA	NA	5.4E-05	(Garriock et al., 2010)	–	–	–	–
							Response	SSRI	Responder	883/608	Mixed	NA	NA	4.4E-05	(Garriock et al., 2010)	–	–	–	–
2	rs6966038 [2]	*UBE3C*	7:157087704	SNV	A/G	0.25 (1000 Gen­ome)	Response	SSRI	Responder	840/837	Mixed	NA	0.7525	.00056	(Cocchi et al., 2016)	–	–	–	–
										883/608	Mixed	NA	1.64 [1.35-1.99]	4.7E-07	(Garriock et al., 2010)	–	–	–	–
							Remission	SSRI	Remitter	575/1102	Mixed	NA	0.7083	3.4E-05	(Cocchi et al., 2016)	–	–	–	–
										743/608	Mixed	NA	1.68 [1.37-2.04]	3.6E-07	(Garriock et al., 2010)	–	–	–	–
GWAS & Candidate Variants																			
1	rs6127921 [2]	*BMP7*	20:57063694	SNV	A/C	0.21 (1000 Geno­­me)	Response: ≥50% reduction in HAMD score after treatment	SSRI	Responder	129/95	Japanese	A	0.59 [0.39-0.88]	.0098	(Esaki et al., 2013)	–	–	–	–
							Response: QIDS	SSRI	NA	883/608	Mixed	NA	0.61 [0.49-0.75]	3.5E-06	(Garriock et al., 2010)	–	–	–	–
							Remitter: QIDS	SSRI	NA	743/608	Mixed	NA	0.57 [0.46-0.72]	1.1E-06		–	–	–	–

Abbreviations: MAF, minor allele frequency;OR, odds ratio; CI, confidence interval; P, p value; PPV, positive predictive value; SNV, single nucleotide variant; bp, base pair, ALFA, allele frequency aggregator; L/S, long/short; MADRS, Montgomery–Åsberg Depression Rating Scale; HAMD and HDRS, hamilton depression rating scale; QIDS, quick inventory of depressive symptomatology; SSRI, selective serotonin reuptake inhibitor; SNRI, serotonin–norepinephrine reuptake inhibitor; TRD, treatment-resistant depression; TCA, tricyclic antidepressant; NA, not available

^a^ Self-calculated values.

^b^Based on literature as not available on NCBI database.

**Table 4.** List of All Reproducible Genes in GWAS Studies, Candidate Studies, and in Both Related to Antidepressant Response

S. No.	Gene	Name	Location	Drug class	Phenotype	Sample size (responder/non-responder)	Population	References	Function
Candidate genes									
1	*SLC6A4*	Solute carrier family 6 member 4	17q11.2	SSRIs	Non-responder	65/83	South Indian	(Mandal et al., 2020)	Monoamine transport
				SSRIs	Non-responder	59/33	Mexican	(Camarena et al., 2019)	
						44/22			
				SSRIs	Non-responder	80/93	Majority Caucasians	(Calabro et al., 2018)	
				SSRIs	Non-remitter	32/14	Caucasian	(Gasso et al., 2018)	
				SSRIs	Non-responder	56/46	South Indian	(Manoharan et al., 2016)	
				SSRIs	Non-responder	164/70	Caucasians	(Seripa et al., 2015)	
				SSRIs	Non-responder	154/85	Korean	(Lim et al., 2014)	
				Mixed	Non-responder	63/34	Italian	(Matsumoto et al., 2014)	
				Atypical	Non-responder	100/183	Korean	(Chang et al., 2014)	
				SSRIs	Non- responder	59/56	Korean	(Won et al., 2012)	
				SSRIs	Non- responder	46/42	Korean	(Myung et al., 2013)	
				SNRIs	Non- responder	54/27	Korean	(S. H. Lee et al., 2010)	
				SSRIs	Non-remitter	29/56	Finnish/Caucasian	(Illi et al., 2011)	
				SSRIs	Non- responder	37/22	Japanese	(Umene-Nakano et al., 2010)	
				SSRIs	Non- responder	243/119	Han Chinese	(Min et al., 2009)	
					Non-remitter	156/206			
				SSRIs	Non-remitter	16/11	Caucasians	(Alexopoulos et al., 2009)	
				Mixed	TRD	98/30	Caucasian	(Wilkie et al., 2009)	
				Atypical	Non- responder	62/39	Korean	(R. H. Kang et al., 2007)	
				TCAs	Non- responder	55/34	Korean	(H. Kim et al., 2006)	
				SSRIs		69/50			
				SSRIs	Non- responder	68/12	Japanese	(Kato et al., 2006)	
				SSRIs	Non- responder	65/65	Croatian	(Bozina et al., 2008)	
				SSRIs	Non- responder	81/143	Han Chinese/ Taiwanese	(Hong et al., 2006)	
				SSRIs	Non-remitter	22/49	Korean	(Choi et al., 2005)	
				SSRIs	Non- responder	77/19	Mixed	(Kraft et al., 2005)	
				SSRIs	Non-remitter	91/40	Spanish	(Arias et al., 2003)	
				SSRIs	Non- responder	40/81	Chinese	(Yu et al., 2002)	
				SSRIs	Non-responder	35/19	Japanese	(Yoshida et al., 2002)	
				SSRIs	Non-responder	87/33	Korean	(D. K. Kim et al., 2000)	
				SSRIs	Non-responder	65/39	Iranian	(Sahraian et al., 2013)	
2	*HTR2A*	5-hydroxytryptamine receptor 2A	13q14.2	SSRIs	Non-remitter	32/14	Caucasian	(Gasso et al., 2018)	Serotonergic neurotransmission
				SSRIs	Non-remitter	NA	Han Chinese	(Su et al., 2016)	
				Mixed	TRD	58/74	European	(Noro et al., 2010)	
				SNRIs and SSRIs	Non-responder	150/115	Japanese	(Kishi et al., 2010)	
					Non-remitter	103/162			
				Mixed	Non-remitter	61/125	Caucasian/Northern Germany	(Lucae et al., 2010)	
				SSRIs	Non-responder	30/46	Caucasian	(Wilkie et al., 2009)	
					Non-remitter	21/51			
				SSRIs	Non-responder	675/270	Mixed (White, Black, and other)	(Paddock et al., 2007)	
					Non-remitter	NA			
				SSRIs	Non-remitter	22/49	Korean	(Choi et al., 2005)	
				SSRIs	Non-responder	57/39	Mixed	(Peters et al., 2004)	
3	*TPH2*	Tryptophan hydroxylase 2	12q21.1	SSRIs & SNRIs	Non responder	205/76	Han Chinese	(Xu et al., 2016)	Biosynthesis of serotonin
				SSRIs	Non remission		Han Chinese	(Su et al., 2016)	
				SSRIs	Non-responder	154/85	Korean	(Lim et al., 2014)	
				Mixed	Non-responder	224/84	Han Chinese	(Xu et al., 2012)	
				SSRIs	Non-responder	126/61	Han Chinese/Taiwanese	(Tsai et al., 2009b)	
				Mixed	Non-responder	84/99	Majority Germans/Caucasians	(Tzvetkov et al., 2008)	
				SSRIs	Non-responder	57/39	Mixed	(Peters et al., 2004)	
4	*BDNF*	Brain derived neurotrophic factor	11p14.1	SSRIs	Non-responder	108/52	Caucasian	(El-Hage et al., 2015)	Promotes neuronal survival in the adult brain
				SSRIs	Non-responder	219/79	Chinese	(X. C. Wang et al., 2014b)	
				Mixed	Non-responder	71/117	European	(Kocabas et al., 2011)	
				SNRIs	Non-responder	23/39	Han Chinese (Taiwanese)	(Chi et al., 2010)	
				SSRIs and SNRIs	Non-remitter	82/191	Caucasoid/Spanish	(Gratacos et al., 2008)	
				SSRIs	Non-responder	57/26	Korean	(Choi et al., 2006)	
5	*HTR1A*	5-hydroxytryptamine receptor 1A	5q12.3	Mixed	Non-responder	224/84	Han Chinese	(Xu et al., 2012)	Serotonergic neurotransmission; G protein-coupled serotonin receptor activity
				Mixed	TRD	58/75	European	(Noro et al., 2010)	
				SSRI, SNRI	Non-responder	22/115	Japanese	(Kato et al., 2009)	
					Non-remitter	78/59			
				SSRI	Non-responder	83/139	Han Chinese/ Taiwanese	(Yu et al., 2006)	
				SSRI	Non-responder	81/143	Han Chinese/ Taiwanese	(Hong et al., 2006)	
				SSRI	Non-responder	35/17	Japanese	(Suzuki et al., 2004)	
6	*ABCB1*	ATP binding cassette subfamily B member 1	7q21.12	SSRI	Non-responder	27/34	Caucasian (Slovakian)	(Vancova et al., 2018)	Transportation of various molecules across extra- and intra-cellular membranes; involved in multidrug resistance
				SSRI	Non-responder	220/70	Han Chinese	(Huang et al., 2013)	
				SSRI	Non-remitter	48/26	Han Chinese	(K. M. Lin et al., 2011)	
				TCA and SSRI	Non-remitter	142	Mexican-Americans	(Dong et al., 2009)	
				SSRI & TCA	Non-responder	147	Mexican Americans	(Wong et al., 2008)	
7	*GRIK4*	Glutamate ionotropic receptor kainate type subunit 4	11q23.3	SNRI	Non-responder	146/29	Han Chinese	(Q. Sun et al., 2019)	Glutamate transmission via activation of ligand-gated ion channels and G protein-coupled membrane receptors
				SSRI	Non-responder	154/85	Korean	(Lim et al., 2014)	
				SSRI,SNRI	Non-responder	205/76	Han Chinese	(Pu et al., 2013)	
				SSRI	Non-responder	260/525	White and African American	(Fabbri et al., 2013)	
				SSRI	Non-responder	675/260	Mixed (White, Black, and other)	(Paddock et al., 2007)	
					Non-remitter				
8	*SLC6A2*	Solute carrier family 6 member 2	16q12.2	SNRI	Non-remitter	76/86	Han Chinese	(Yeh et al., 2015)	Regulates norepinephrine homeostasis
				TCA	Non-remitter		MexicanAmericans	(Dong et al., 2009)	
				SSRI	Non-remitter				
				TCAs	Non-responder	55/34	Koreans	(H. Kim et al., 2006)	
				SNRI	Non-responder	50/30	Japanese	(Yoshida et al., 2004)	
9	*COMT*	Catechol-O-methyltransferase	22q11.21	SSRI	Non-remitters	24/35	Japanese	(Fukui et al., 2014)	Catalyzes the transfer of a methyl group from S-adenosylmethionine to catecholamines (dopamine, epinephrine, and norepinephrine)
				SSRIs	Non-remitters	691/541	White non-Hispanic	(Y. Ji et al., 2012)	
				Mixed	TRD	73/294	Caucasian	(Kocabas et al., 2010)	
						61/105			
				SSRI	Non-remitters	24/35	Japanese	(Fukui et al., 2014)	
				SSRI	Non responder	101/52	Han Chinese	(Tsai et al., 2009a)	
10	*GNB3*	G protein subunit beta 3	12p13.31	SSRI	Non-responder	33/67	Iranian	(D. Firouzabadi et al., 2019)	Integrate signals between receptors and effector proteins
				SSRIs and SNRI	Non-responder	35/66	Han Chinese/Taiwanese	(E. Lin et al., 2009a)	
				Mixed	Non-responder	58/105	Caucasian	(Wilkie et al., 2007)	
					Non-remitters	70/40			
				Mixed	Non-responder	67/39	Korean	(H. J. Lee et al., 2004)	
11	*CRHR1*	Corticotropin releasing hormone receptor 1	17q21.31	Atypical	Responder	1298/1734	French (Caucasian)	(Ramoz et al., 2020)	G protein-coupled receptor activity; major regulator of the hypothalamic-pituitary-adrenal pathway
					Remission				
				SSRI	Non-responder	37/108	Chilean	(Ventura-Junca et al., 2014)	
				SSRI, SNRI	Non-remitter	150/123	Han Chinese	(L. Y. Geng et al., 2014)	
12	*GRM7*	Glutamate metabotropic receptor 7	3p26.1	SNRI	Non-responder	146/29	Han Chinese	(Q. Sun et al., 2019)	Glutamatergic neurotransmission
				SSRI	Non-responder	260/525	White and African-American	(Fabbri et al., 2013)	
					Non-responder	189/352	White non-Hispanic		
13	*CYP1A2*	Cytochrome P450 family 1 subfamily A member 2	15q24.1	SNRI	Non-responder	97/78	Han Chinese	(Zhu et al., 2019)	Involved in drug metabolism and synthesis of cholesterol, steroids and other lipids
				SSRI	Non-remitters	69/102	Han Chinese	(K. M. Lin et al., 2010)	
14	*CLOCK*	clock circadian regulator	4q12	SSRIs	Non-responder	284/47	Han Chinese	(H. Y. Ma et al., 2019)	Regulation of circadian rhythms
				SSRIs	Non-responder	60/61	Japanese	(Kishi et al., 2009a)	
					Non-remitter	45/76			
15	*NR3C1*	Nuclear receptor subfamily 3 group C member 1	5q31.3	SSRIs	Non-responder	70/30	Caucasian (Iranian)	(Nouraei et al., 2018)	Involved in inflammatory responses, cellular proliferation, and differentiation in target tissues
				SSRIs	Non-responder	104/56	Japanese	(Takahashi et al., 2014)	
16	*CRHR2*	Corticotropin releasing hormone receptor 2	7p14.3	TCA	Non-responder		Mexican Americans	(Wong et al., 2008)	G protein-coupled receptor activity; major regulator of the hypothalamic-pituitary-adrenal pathway
				SSRI	Non-responder	97/51	Spanish	(Papiol et al., 2007)	
17	*FKBP5*	FKBP prolyl isomerase 5	6p21.31	SSRIs	Non-responder	954/416	Mixed (White non-Hispanic, Black)	(Lekman et al., 2008)	Role in immunoregulation; involving protein folding and trafficking
					Non-remitter	723/467	Mixed (White non-Hispanic, Black)		
				Mixed	Non-responder	147/86	German/Caucasian	(Binder et al., 2004)	
18	*TPH1*	Tryptophan hydroxylase 1	11p15.1	SSRIs	Non-remitter	61/44	Korean	(Ham et al., 2007)	Biosynthesis of serotonin
				SSRIs	Non-responder	35/42	Italian	(Serretti et al., 2001)	
GWAS genes									
1	*CNTN5*	Contactin 5	11q22.1	Mixed	Responder vs non-responder	61/31	Japanese	(Sasayama et al., 2013)	Role in the formation of axon connections in the developing nervous system
				Dissociative anesthetics (S-ketamine)	Remission vs non-remission	255/272	European	(Li et al., 2020)	
2	*LHFPL3*	LHFPL tetraspan subfamily member 3	7q22.2-q22.3	Dissociative anesthetics (S-ketamine)	Remission vs non-remission	255/272	European	(Li et al., 2020)	Sensory perception of sound
				Remission	SSRI	743/608	African-American and Caucasian	(Garriock et al., 2010)	
3	*AGBL1*	AGBL carboxypeptidase 1	15q25.3	Dissociative anesthetics (S-ketamine)	Remission vs non-remission	255/272	European	(Li et al., 2020)	Protein side chain deglutamylation
				SSRI	Remission	840/837	Mixed	(Cocchi et al., 2016)	
4	*CRADD*	CASP2 and RIPK1 domain containing adaptor with death domain	12q22	Dissociative anesthetics (S-ketamine)	Remission	255/272	European	(Li et al., 2020)	Acts in promoting apoptosis
				SSRI	Remission	42/67	Korean	(Cocchi et al., 2016)	
5	*LARGE1*	LARGE xylosyl- and glucuronyltransferase 1	22q12.3	Dissociative anesthetics (S-ketamine)	Response	255/272	European	(Li et al., 2020)	
				Remission	SSRI	743/608	African-American and Caucasian	(Garriock et al., 2010)	
6	*Y_RNA*	Ro60, Y RNA binding protein	1q31.2	Dissociative anesthetics (S-ketamine)	Remission	255/272	European	(Li et al., 2020)	Enables RNA binding activity
				SSRI	Remission	263/218	Korean	(Myung et al., 2015)	
7	*MYO5B*	Myosin VB	18q21.1	Dissociative anesthetics (S-ketamine)	Response	255/272	European	(Li et al., 2020)	Involved in vesicular trafficking via its association with the CART complex
				SSRI	Response	263/218	Korean	(Myung et al., 2015)	
8	*ARHGAP8*	Rho GTPase activating protein 8	22q13.31	Dissociative anesthetics (S-ketamine)	Remission	255/272	European	(Li et al., 2020)	Regulate cell processes involved in cytoskeletal changes
				SSRI	Remission	42/67	Korean	(Cocchi et al., 2016)	
9	*CFAP61*	Cilia and flagella associated protein 61	20p11.23	SSRI	Remission	840/837	Mixed	(Cocchi et al., 2016)	Involved in cilium movement and cilium organization
				SSRI	Responder vs non-responder	883/608	African-American and Caucasian	(Garriock et al., 2010)	
					Remitter vs non-responders	743/608			
10	*MTCL1*	Microtubule crosslinking factor 1	18p11.22	SSRI	Response	416/449	Mixed	(Biernacka et al., 2015)	Enables microtubule binding activity
				SSRI	Response	263/218	Korean	(Myung et al., 2015)	
11	*AUTS2*	Activator of transcription and developmental regulator AUTS2	7q11.22	SSRI	Response	840/837	Mixed	(Cocchi et al., 2016)	Act as key regulator of the transcriptional network during brain development
				SSRI	Response	263/218	Korean	(Myung et al., 2015)	
				SSRI	Response	118/112			
				TCA	Response	116/43			
GWAS and candidate genes									
1	*COMT*	Catechol-O-methyltransferase	22q11.21	SSRI	Non-remitters	24/35	Japanese	(Fukui et al., 2014)	Catalyzes the transfer of a methyl group from S-adenosylmethionine to catecholamines (dopamine, epinephrine, and norepinephrine)
				SSRIs	Non-remitters	691/541	White non-Hispanic	(Y. Ji et al., 2012)	
				Mixed	TRD	73/294	Caucasian	(Kocabas et al., 2010)	
						61/105			
				SSRI	Non-remitters	24/35	Japanese	(Fukui et al., 2014)	
				SSRI	Non responder	101/52	Han Chinese	(Tsai et al., 2009a)	
				Mixed	Non-remitters	119/36	South Korea	(H. J. Kang et al., 2020)	
2	*PDLIM5*	PDZ and LIM domain 5	4q22.3	SSRI	Non-responder	98/87	Han Chinese	(Z. Liu et al., 2013a)	Function in cardiomyocyte expansion and in restraining postsynaptic growth of excitatory synapses
				Mixed	Response	61/31	Japanese	(Sasayama et al., 2013)	
3	*NR3C2*	Nuclear receptor subfamily 3 group C member 2	4q31.23	SSRI	Response	840/837	Mixed	(Cocchi et al., 2016)	Mineralcorticoid receptor; Inhibitory control of HPA axis; memory
				SNRI	Non-remission	94/75	Han Chinese	(Yuan et al., 2020)	
4	*HTR2A*	5-hydroxytryptamine receptor 2A	13q14.2	SSRI	Response	840/837	Mixed	(Cocchi et al., 2016)	Serotonergic neurotransmission
				SSRIs	Non-remitter	32/14	Caucasian	(Gasso et al., 2018)	
				SSRIs	Non-remitter	NA	Han Chinese	(Su et al., 2016)	
				Mixed	TRD	58/74	European	(Noro et al., 2010)	
				SNRI and SSRIs	Non-responder	150/115	Japanese	(Kishi et al., 2010)	
					Non-remitter	103/162			
				Mixed	Non-remitter	61/125	Caucasian/Northern Germany	(Lucae et al., 2010)	
				SSRIs	Non-responder	30/46	Caucasian	(Wilkie et al., 2009)	
					Non-remitter	21/51			
				SSRIs	Non-responder	675/270	Mixed (White, Black, and other)	(Paddock et al., 2007)	
					Non-remitter	NA			
				SSRIs	Non-remitter	22/49	Korean	(Choi et al., 2005)	
				SSRIs	Non-responder	57/39	Mixed	(Peters et al., 2004)	

Abbreviations: SSRI, selective serotonin reuptake inhibitor; SNRI, serotonin–norepinephrine reuptake inhibitor; TRD, treatment-resistant depression; TCA, tricyclic antidepressant; NA, not available.

### Functional Annotation

The SNPs were mapped to genes using National Center for Biotechnology Information (NCBI) dbSNP following the GRCh38.p13 genome assembly. To predict the functional consequences of the included genetic variants, we used different in silico prediction tools to assess the deleterious effect of the amino acid change in case of a missense variant and the regulatory potential of intronic SNPs (Kaur et al., 2014). The different tools used for function prediction were SIFT (Ng and Henikoff, 2001), PolyPhen2 (Adzhubei et al., 2010), RegulomeDB 2.0.3 (Boyle et al., 2012), and SNP info (Xu and Taylor, 2009). Additionally, we checked the genes of these replicated variants: (1) for which behavior/neurological phenotype was observed in knockout mice and the phenotype data were extracted from the Mouse Genome Informatics database (http://www.informatics.jax.org/); (2) assessed whether the gene is a known target of an antidepressant drug as detailed in the drug–gene interaction database (www.DGidb.com); and (3) whether the gene is preferentially expressed in the brain means the average expression in all brain tissues was higher than the average expression in non-brain tissues. This was assessed by using gene expression data from all 53 tissues of the Gene-Tissue expression Consortium. Data obtained from each tool have been described in supplementary Tables 5 and 6.

### Assessment of Diagnostic Predictability of Genetic Variants

The diagnostic predictability of genetic variants indicates its ability to precisely predict the occurrence of a disease phenotype in patients carrying the risk allele. We attempted to evaluate the diagnostic predictability for genetic variants with more than 1 publication of disease-variant association as well as response-variant association. True positives (TP) and false negatives (FN) are values representing the risk allele and wild-type allele carrier, respectively, in cases. Conversely, true negatives (TN) and false positives (FP) are defined as wild-type and risk allele carriers, respectively, in controls. The TP rate or sensitivity is calculated as ΣTP/(ΣTP + ΣFN) and specificity is ΣTN/(ΣTN + ΣFP). Furthermore, the positive predictive values are calculated as ΣTP/(ΣTP + ΣFP).

## RESULTS

### Literature Search

#### Acquisition of MDD Risk–Associated Studies—

**Figure 1.**
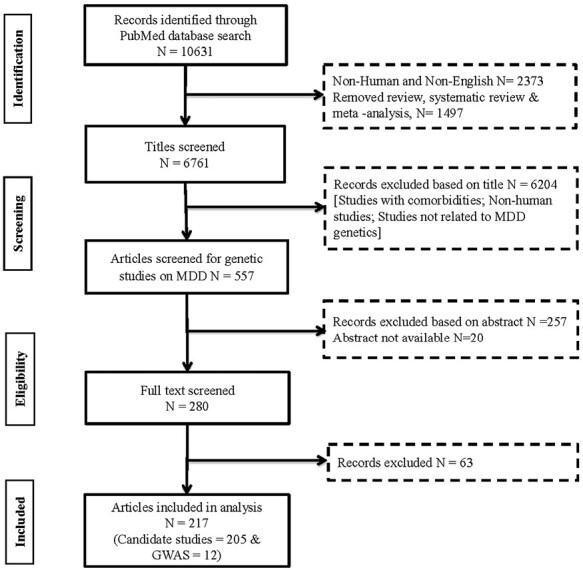
Identification and selection process of relevant genetic association studies of major depressive disorder (MDD).

A systematic search strategy for identifying MDD associated genes extracted 10 631 genetic association studies, which were further reduced to 280 articles of relevance after title and abstract screening. Among these excluded articles, 2382 non-human/other language, 6081 other disorder/phenotype/comorbidity, 201 association study of MDD with other phenotype, 120 no association, and 1567 other articles including non-genetic studies, review articles, editorials, meta, comments, and letters were also removed. The remaining 280 articles were then searched for their full text, and 63 articles were again excluded from the study because they did not meet the inclusion criteria, leading to a final of 217 articles for data extraction and processing. Twelve of these were GWAS studies and 205 were candidate association studies (Figure 1).

#### Acquisition of Antidepressant Response–Associated Studies—

**Figure 2.**
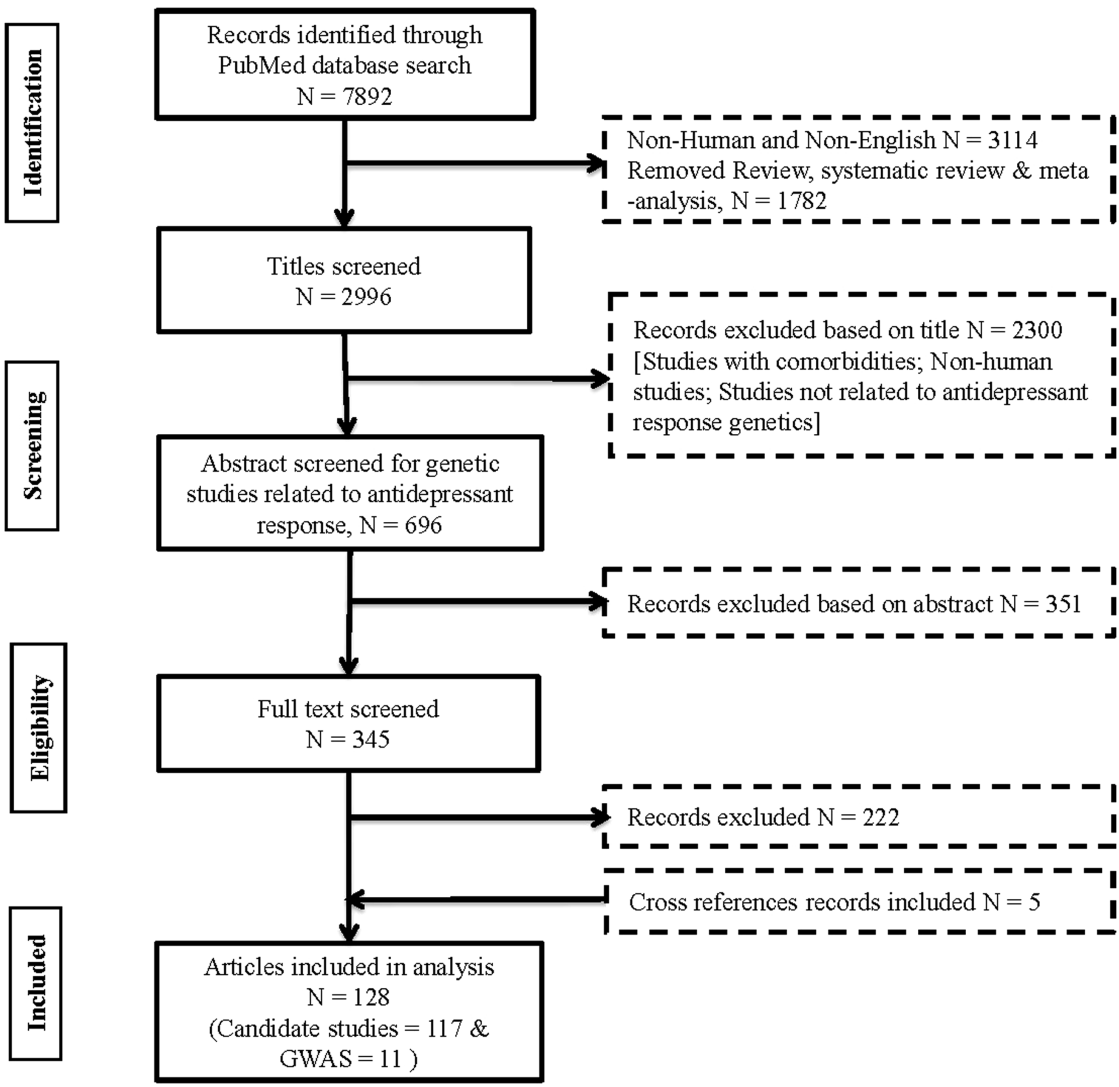
Identification and selection process of relevant genetic association studies of antidepressant response.

Similarly, a systematic search strategy for identifying antidepressant response associated genes extracted 7892 genetic association studies, which was further reduced to 345 articles of relevance after title and abstract screening. Among these excluded articles, 3109 were non-human, 160 were other language, 1768 were not an antidepressant response study, 533 were other disorders, 39 were methylation/miRNA, protein-related studies, 82 no association studies and 1856 other articles including non-genetic studies, review articles, genome-wide studies, editorials, and letters were also removed. The remaining articles were then searched for their full text and 222 articles were again excluded from the study, as they did not meet the inclusion criteria, leading to a final of 128 articles (including 5 articles that are additionally added through cross-references) for data extraction and processing. Of these, 11 were GWAS response studies and 117 were candidate response association studies (Figure 2).

### Study Characteristics

#### Candidate Genetic Association Studies Related to MDD Risk—

We have summarized the main characteristics of the identified studies in supplementary Table 1. Only significant *P* values and ORs are presented. The 205 articles reported nominal significant associations (*P* < .05). These 205 articles reported significant results for 378 polymorphisms in 156 unique genes. A total of 196 339 individuals (n = 80 891 cases and n = 115 448 controls) were included in all the studies. The 27 genetic variants were confirmed by at least 2 studies. The number of patients ranged from 11 to 1738, and their age varied from 14 to 102 years. The ratio of male to female was 1.30 and 1.35 in cases and controls, respectively. Of all the candidate association studies, 90 studies were performed in East Asia, 87 in Europe, 11 in America, 7 in the Caucasian population, 4 in Middle Eastern countries, 3 in South Asia, 2 included mixed population, 1 from African population, 1 involving white participants and 1 with white non-Hispanic individuals. The most used MDD diagnostic criterion was based on the DSM (n = 164), followed by The International Classification of Diseases (ICD) (n = 21), studies that used both DSM and ICD criteria (n = 7), and others (n = 11).

#### GWAS Studies Related to MDD Risk—

A total of 12 GWAS determine the association of genetic variants in MDD. These 12 articles reported significant results for 819 polymorphisms in 387 genes. In total, 268 481 patients with MDD and 841 656 controls were included. The mean age of the participants was 45.55 years for MDD cases and 49.71 years for controls. The overall ratio of male to female was 1.12. Most of the studies were performed in the European population (n = 9), Han Chinese (n = 1), and mixed population (n = 2). The MDD patients were diagnosed based on Structured Clinical Interview fulfilling DSM/ICD criteria (n = 11), although in 1 study self-reported MDD patients were also recruited. The majority of studies included patients with MDD/unipolar depression/recurrent depression (n = 10), except in 2 studies, where 1 included recurrent early-onset MDD patients and in another broad depression and probable MDD patients were recruited in the study. All studies employed an array-based genotyping method. The characteristics of the included studies and genotypic/allelic distributions of the polymorphisms are shown in supplementary Table 2.

#### Candidate Genetic Association Studies Related to Antidepressant Response—

A total 116 articles reported nominal significant associations (*P* < .05). The characteristics of the included studies and genotypic/allelic distributions of the polymorphisms are shown in supplementary Table 3. The table includes only those studies that were either in the form of responder vs non-responder (n = 64) or remitter vs non-remitter (n = 34). Thirteen studies examined both response as well as remission status. Additionally, 6 studies checked the association in treatment-resistant patients. A total of 21 753 (12 577 responders and 9029 non-responders) and 11 299 (4546 remitters and 4906 non-remitters) patients were included in the studies. The recruited cohort were of East Asian (55.17%), European (27.58%), American (7.75%), and other (9.48 %) origin. All the studies recruited their patients based on either DSM (n = 112) or ICD-10 (n = 4) diagnostic criteria. Remission was defined as a final Hamilton Rating Scale for Depression (HAM-D) or Montgomery and Åsberg Depression Rating Scale (MADRS) total score of 7 or less, and response was defined as at least a 50% decrease in HAM-D or MADRS total score. Maximum studies have employed HAM-D scale (n = 69), followed by Quick Inventory of Depressive Symptomatology (QIDS) (n = 5), MADRS (n = 2), and Beck’s Depression Inventory (n = 1) for accessing the response in patients. Similarly, for remission assessment HAMD/ MADRS scale (n = 39) and QIDS (n = 5) were employed on the patients. Furthermore, we observed the majority of studies were based on selective serotonin reuptake inhibitors (SSRIs) (n = 69) followed by mixed therapy (n = 33), serotonin and norepinephrine reuptake inhibitors (n = 9), atypical (n = 3), and tricyclic antidepressants (n = 2). Of these, 60 studies were on monotherapy. There were some studies (n = 6) that determined the response association with multi-drug therapy and further also examined the association based on monotherapy. The follow-up period in all the studies ranged from 2 to 18 weeks. In total, 97 unique genes and 217 variants were found to be significantly associated with antidepressant response.

#### GWAS Studies Related to Antidepressant Response—

A total of 11 studies have examined association between SNPs and antidepressant response (supplementary Table 4). There were 3 response studies, 3 remission studies, and 5 associated with both response and remission status. A total 39 533 patients (23 141 responder and 11 322 non-responders; 2392 remitter and 1413 non-remitter) were included in either a discovery or replication cohort of all the studies. Included studies were performed in different ethnic populations, from Korean (n = 3), European (n = 2), Japanese (n = 1), Mexican American (n = 1), White non-Hispanic (n = 1), Caucasian (n = 1), and Mixed (n = 3). The male to female ratio in response studies was 1.32 and in remission studies was 0.79. We could not determine the mean age inof all the studies due to the heterogeneity in data reporting as well as the unavailability of raw data in some of the studies. All studies employed the DSM/ICD-10 diagnostic criteria to determine the MDD phenotype except 1 study, which utilized a questionnaire designed by 23andMe to recruit self-reported patients. These 11 studies reported significant results for 704 polymorphisms in 315 genes. Only rs1908557 SNPs have shown genome-wide significance with a *P* value of 2.6 × 10^−8^.

### Reproducibility of Findings

**Figure 3.**
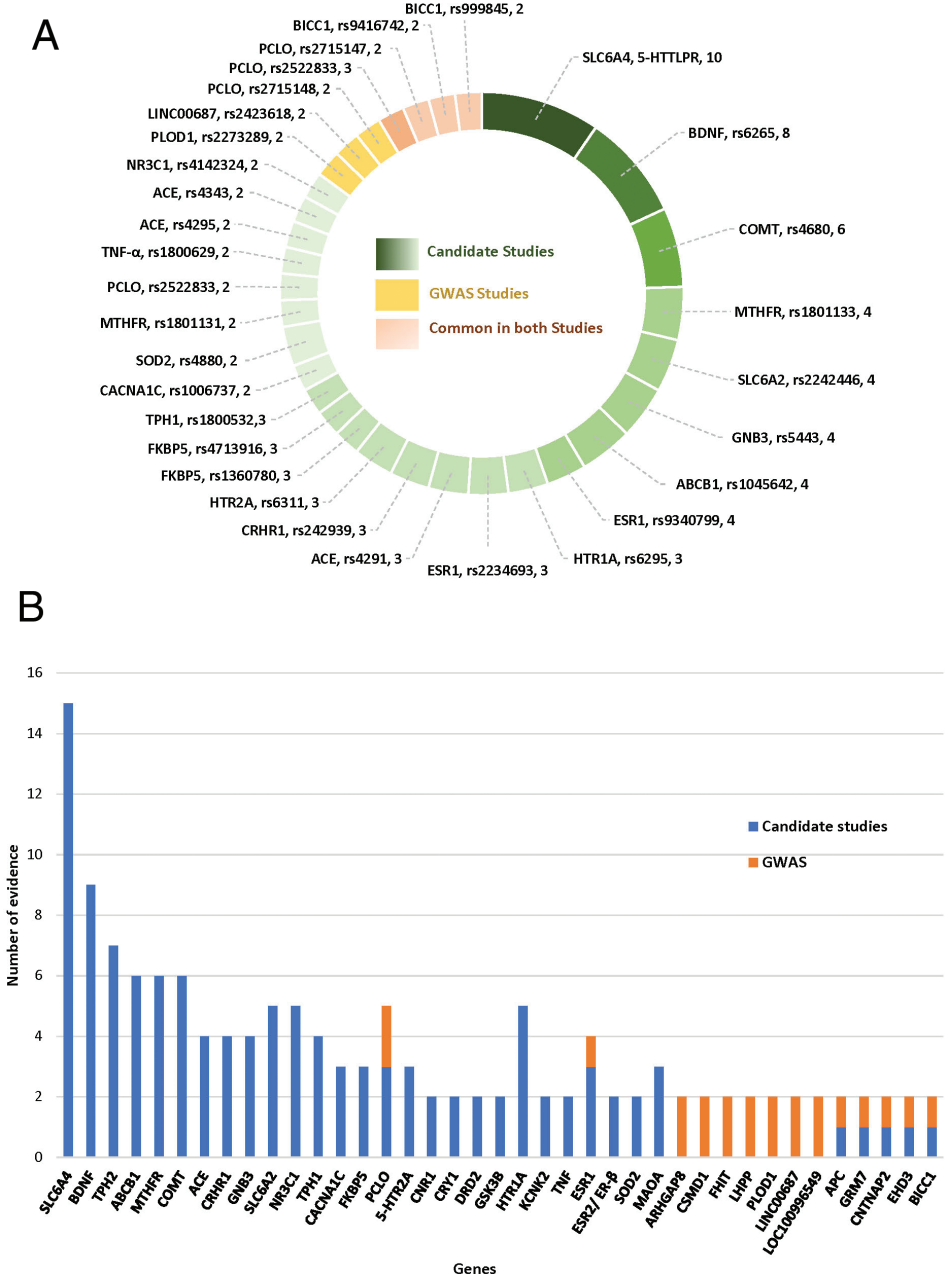
(A) Significantly associated reproducible* genetic variants: 24 in candidate studies, 3 in genome-wide studies, and 4 variants that are common in both candidate and genome-wide association studies (GWAS). Color represents the studies; gradient represents the no. of studies/evidence. Label order: gene, SNPs, No. of studies/evidence. *Minimum in 2 studies. (B) Reproducible genes from genome-wide and candidate association studies: 27 in candidate studies, 8 in genome-wide studies, and 7 variants that are common in both candidate and GWAS studies.

To narrow the focus of the results presented above, we chose to concentrate our analysis on the replicated genetic variants and genes that were reported 2 or more times across studies. Of all the positive associations, we found a total of 34 replicated variants with MDD susceptibility. However, 3 variants (rs120074175/TPH2, rs6195/NR3C1, and rs6189+rs6190/NR3C1) had MAF <0.05 and hence were removed from further analysis. The remaining 31 replicated variants from candidate studies (n = 24), GWAS (n = 3), and both candidate and GWAS studies (n = 4) are shown in Table 1 and Figure 3A. Additionally, information related to the number of studies reporting no association between these variants and MDD susceptibility is provided in supplementary Table 7A. Further, 5-HTTLPR/*SLC6A4* was the genetic factor most frequently investigated (n = 10), followed by rs6265/*BDNF* (n = 8), rs4680/*COMT* (n = 6), rs1801133/*MTHFR* (n = 4), and others. Interestingly, of all 31 replicated variants, we observed 13 variants (3 from GWAS, 7 from candidate, and 3 from both), that is, C allele of rs2273289/*PLOD1* in 2 studies, C allele of rs2715148/*PCLO* in 2 studies, C allele of rs2423618/*LINC00687* in 2 studies, T allele of rs1801133/*MTHFR* in 4 studies, T allele of rs5443/*GNB3* in 4 studies, G allele of rs242939/*CRHR1* in 3 studies, G allele of rs6295/*HTR1A* in 3 studies, A allele of rs1006737/*CACNA1C* in 2 studies, T allele of rs4880/*SOD2* in 2 studies, C allele of rs1801131/*MTHFR* in 2 studies, A allele of rs2715147/*PCLO*, G allele of rs9416742/*BICC1*, and A allele of rs999845/*BICC1* showed consistency in reporting the risk allele, whereas 18 variants had inconsistent risk alleles across studies. Moreover, we also checked the reproducibility of genes where 27 genes in candidate studies, 8 genes in GWAS, and 7 genes in both candidate and GWAS studies were replicated, which is represented in Table 2 and Figure 3B.

**Figure 4.**
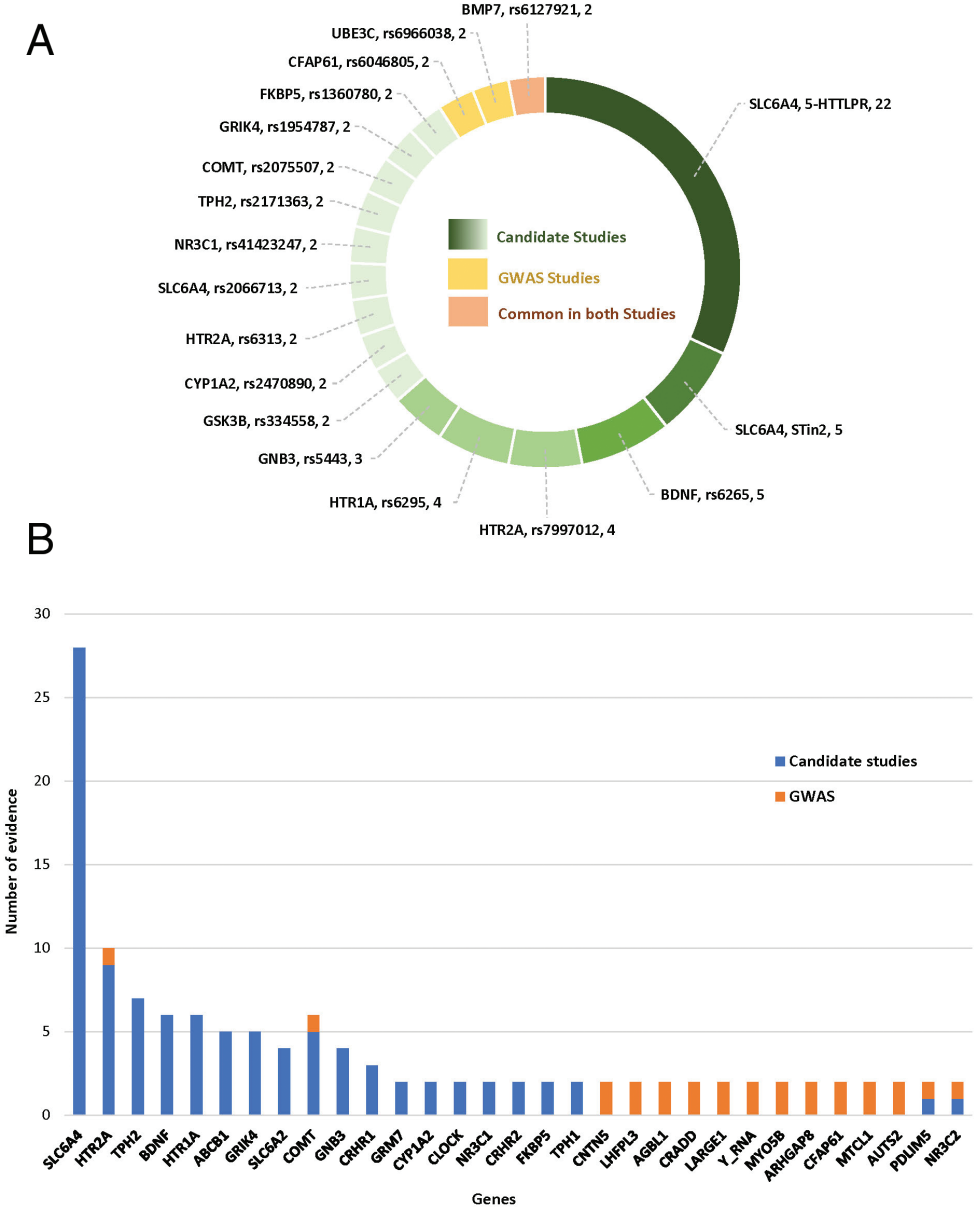
(A) Significantly associated reproducible* genetic variants: 15 in candidate studies, 2 in genome-wide studies, and 1 variant that is common in both candidate and genome-wide association studies (GWAS). Color represents the studies; gradient represents the no. of studies/evidence. Label order: gene, SNPs, No. of studies/evidence. *Minimum in 2 studies. (B) Reproducible genes from genome-wide and candidate drug response association studies: 18 in candidate studies, 11 in genome-wide studies, and 4 variants that are common in both candidate and GWAS studies.

On the other hand, we found 15 genetic variants in candidate, 2 in GWAS, and 1 common in candidate and GWAS studies for drug response, as shown in Table 3 and Figure 4A, whereas information related to the number of studies reported no association with the antidepressant response is provided in supplementary Table 7B. SNPs located in *SLC6A4*, *BDNF*, *HTR2A*, *HTR1A*, *GNB3*, *CYP1A2*, *NR3C1*, *TPH2*, *COMT*, *GRIK4*, *FKBP5*, *CFAP61*, and *UBE3C* were positively replicated in independent populations. Moreover, the only variant that showed a positive association in both GWAS and candidate studies was rs6127921 of *BMP7* gene. In addition, 18 genes in candidate studies, 11 in GWAS, and 4 genes in both candidate and GWAS studies were found to be replicated in response studies, as represented in Table 4 and Figure 4B.

### Functional Annotation of Reproducible Findings

This study further sought to explore the molecular consequences of these reproducible genetic variants (missense and non-coding) from MDD susceptibility and antidepressant response studies by using different computational methods. The different functionality prediction servers such as SIFT, PolyPhen-2, RegulomeDB, and SNP info were utilized. SIFT and PolyPhen-2 sieved the deleterious non-synonymous SNPs, followed by RegulomeDB, which provides information about regulatory SNPs. The prediction analysis of the effect of SNPs on specific functions, including splicing regulations, miRNA binding site, regulatory potential, and conserveness, was performed using the FuncPred tool of SNPinfo. Consequently, we checked a total of 31 replicated variants (7 missense, 14 intronic, 6 UTR/upstream, 1 downstream and 3 synonymous) resulting from MDD susceptible studies shown in supplementary Table 5. Here, out of 7 missense SNPs, SIFT predicted 1 SNP (14.2%) as deleterious and the rest 7 (85.7%) as tolerated. PolyPhen-2 predicted 2 SNPs (28.5%) as damaging and 5 SNPs (71.4 %) as neutral or benign. Importantly, only nonsynonymous/missense variant rs1801133/*MTHFR* was predicted to be deleterious and probably damaging by SIFT and PolyPhen-2 respectively. In addition, rs6265/*BDNF* was also predicted as possibly damaging by PolyPhen-2 though tolerated by SIFT. These findings were further strengthened by the ClinVar database as rs1801133 was reported to be pathogenic and rs6265 as a risk factor for other psychiatric disorders such as schizophrenia and bipolar, respectively. Out of all 31 variants, SNPinfo predicted 1 variant (i.e., rs2715148/*PCLO*) affects the miRNA binding site activity and 7 variants (rs4680, rs4880, rs1801131, rs2522833, rs1045642, rs4343, rs2522833) were found to affect the splicing activity. Moreover, 26.6% of SNPs had >80% conservation and 16.6% of SNPs had >40% regulatory potential. Further, among the 31 variants evaluated with RegulomeDB, 25 variants were predicted to have a regulatory effect ranging from 1d to 5. Out of these 25 variants, 2 variants (i.e., rs1800629/*TNF*, score = 1d; and rs1801131/*MTHFR*, score = 1f) got the lowest scores and thus are most likely to be involved in eQTL functions. Similarly, 4 variants (rs4343/*ACE*, rs2242446/*SLC6A2*, rs41423247/NR3C1, rs2273289/*PLOD1*) were assigned a rank of 2b, which indicates TF binding + any motif + DNase Footprint + DNase peak.

We also observed, of 31 replicated variants, 26 (83.8%) and 16 (51.6%) genes were found to have behavioral/neurological knockout mouse model and are known targets of antidepressant drugs, respectively. Further, 12 (38.7%) genes have shown to be preferentially expressed in the brain (supplementary Table 5).

Likewise, we checked the functional relevance of 18 variants (7 intronic, 5 UTR/upstream, 3 synonymous, 1 missense, and 2 unknown) resulted from antidepressant response studies shown in supplementary Table 6. One missense variant, rs6265/*BDNF*, was predicted by PolyPhen-2 to be possibly damaging. Of all genetic variants, SNPInfo predicted none of the SNPs to affect splicing and miRNA binding activity. However, 3 SNPs had >80% conservation, and 3 SNPs had >40% regulatory potential. Further, RegulomeDB analysis identified 15 SNPs with regulatory effect ranging from 1f to 5. Three variants, rs2470890/*CYP1A2* (score = 1f), rs41423247/*NR3C1* (score = 2b), and rs6313/*HTR2A* (score = 3a), were found most likely to affect the transcription factor binding activity with a score <3. In addition, 15 (83.3%) and 13 (72.2%) genes of replicated variants were found to have behavioral/neurological knockout mouse phenotype and known targets of antidepressant drugs, respectively. Also, 13 (72.2 %) genes have been shown to be preferentially expressed in the brain (supplementary Table 6).

### Diagnostic Predictability

The allele frequency of reproducible variants was utilized for sensitivity and specificity analysis (Table 1). The majority of replicated findings were inconsistent in their reported risk allele such as the 5-HTTLPR, which appeared in 10 studies, the short (S) form was reported in 7 studies, and long (L) form in 3 studies. Therefore, studies reported the same type of risk allele were pooled together for sensitivity and specificity analysis. G allele of rs6265/*BDNF* had the highest sensitivity (0.78), whereas G allele of rs242939/*CRHR1* had the highest specificity (0.93). Moreover, the positive predictive values for 19 genetic variants ranged from 0.49 to 0.66. Most of them had moderate PPV. However, we could not perform the diagnostic predictability for a few variants (n = 6) due to unavailable raw data. Additionally, if a replication of variant is due to 2 studies that reported the 2 different risk alleles (n = 6), then assessment of sensitivity and specificity could not be calculated.

In response studies, we could only assess the diagnostic predictability of 5 out of 18 variants (PPV between 0.36 and 0.66) because the majority of replicated findings were inconsistent in their response status, drug class, associated phenotype, population, and reported risk allele and therefore could not be cumulated/pooled (Table 3).

## DISCUSSION

This study comprehensively evaluated the original articles on genetic association studies of MDD susceptibility and antidepressant treatment response, independently. A total of 217 studies of MDD susceptibility and 128 studies of drug response met our a priori inclusion criteria and were described in the present study. The results of this article have shown that the majority of the positive associations were confirmed by only 1 study and therefore cannot exclude the possibility of having been obtained by chance, and thus are not sufficient to establish a link with MDD susceptibility or antidepressant treatment response. Therefore, the rest of the discussion is limited to reproducible findings at the SNP or gene level generated in more than 1 independent study. In total, we found 31 and 18 replicated variants in MDD susceptibility and antidepressant drug response, respectively. Further, taking all these findings into account, it appears most studies used the candidate gene approach. As a result, a large number of SNPs and genes were found to be replicated in candidate studies compared with GWAS studies, which demonstrate the surprisingly small overlap of genetic variants across studies. Also, none of the variants from candidate studies appeared among the top hits in any of the identified GWAS. Nevertheless, we identified an important overlap of positive association of 4 (rs2522833/*PCLO*, rs2715147/*PCLO*, rs9416742/*BICC1*, and rs999845/*BICC1*) and 1 (rs6127921/*BMP7*) variants between candidate and GWAS studies in MDD susceptibility and antidepressant response, respectively. Replication of variants between candidate and unbiased GWAS studies greatly increases the acceptability of genotype and phenotype association. Besides replication, additional evidence regarding the biological relevance of *PCLO*, *BICC1*, and *BMP7* has also been reported in the literature. A meta-analysis by Hek et al. (2010) provided additional evidence of significant association (*P* = 2.16 × 10^−3^) between SNP rs2522833/PCLO and depressive disorders in a population-based study. In addition, the rs2522833 variant causes change in amino acid from serine to alanine in a piccolo C2A calcium binding domain (Verbeek et al., 2012), whose overexpression causes depression-like behavior in mice (Furukawa-Hibi et al., 2010). Similarly, expression of *BICC1* was reported to be upregulated in the post-mortem brains of individuals with MDD and expression levels of the same observed to be reduced after antidepressant treatment in rat models of chronic stress (Ota et al., 2015). Furthermore, a significant reduction in the expression of the *BMP7* gene in the locus coeruleus region of post-mortem brains of individuals with MDD has also been shown in the literature (Ordway et al., 2012). Thus, these variants would most likely be of considerable interest in future studies.

Further, the functional annotation of reproducible variants of MDD susceptibility revealed 2 non-synonymous/missense variants (rs1801133/*MTHFR* and rs6265/*BDNF*) as deleterious/damaging and 6 variants (rs1800629/*TNF*, rs1801131/*MTHFR*, rs4343/*ACE*, rs2242446/*SLC6A2*, rs2273289/*PLOD1*, and rs41423247/*NR3C1*) with a score of <3 had high regulatory effect in disease condition. On the other hand, functional annotation of variants from antidepressant treatment response predicted 1 non-synonymous/missense variant (rs6265/*BDNF*) as damaging and 3 non-coding variants (rs2470890/*CYP1A2*, rs6313/*HTR2A*, and rs41423247/*NR3C1*) with a high regulatory effect. Moreover, diagnostic predictability of reproducible variants revealed moderate PPV ranges from 0.49 to 0.66 in MDD susceptibility and 0.36 to 0.66 in antidepressant drug response. This quantitative measure offers a substantial hint toward a direction of predictability and also corroborates increasing the significance of replicated findings.

Of all the positive association studies, 5-HTT gene-linked polymorphic region (5-HTTLPR) polymorphism has gained particular attention for being the most replicated variant in both disease (n = 10) and drug response studies (n = 22). The 2 allelic variants, S and L, are due to deletion and insertion of 44 bp in the promoter region, respectively. The S allele has been demonstrated to have lower transcriptional activity than the L allele, resulting in the reduction of serotonin transporter in the cell membrane. Consequently, the reduction of 5-HTT causes the imbalance of serotonin concentration and function (Fratelli et al., 2020). However, we observed a significant inconsistency in the reported risk allele across studies. For example, in disease, 5HTTLPR appeared in 10 studies, wherein the S form was reported as risk allele in 7 studies and the L form in 3 studies. The heterogeneity in reported risk allele even within the same geographical population weakens our confidence in proposing them as a probable genetic diagnostic marker. In a similar line, there were 17 (rs6265, rs4680, rs2242446, rs1045642, rs9340799, rs2234693, rs4291, rs6311, rs1360780, rs4713916, rs1800532, rs1800629, rs4295, rs6195, rs2522833, rs4343, and rs41423247) and 9 (5HTTLPR, rs6265, rs7997012, rs6295, rs5443, rs2470890, rs6313, rs2171363, rs1954787) replicated variants associated with MDD susceptibility and drug response, respectively, where authors have found discrepancy in reported risk allele, suggesting a more robust future study design for such candidate gene studies.

Therefore, reproducible variants reporting a single risk allele across studies are of prime importance and could be taken forward for diagnostic and therapeutic application. In total 14 variants in MDD susceptibility and 6 in antidepressant response reported the consistent findings in terms of risk allele. However, due to the unavailability of raw data, we could assess the diagnostic predictability of only 7 and 2 variants in disease and drug response, respectively. Interestingly, we observed variants of *FKBP5* and *HTR2A* genes are being commercially utilized for diagnostic application in MDD gene panels (Rubinstein et al., 2013), and the replication of variants in these genes, rs1360780/*FKBP5* (PPV = 0.53), rs4713916/*FKBP5* (PPV = 0.53), and rs6311/*HTR2A* (PPV = 0.54), are demonstrated in our analysis. However, the diagnostic predictability of these variants were found to be moderate. This observation inclined our interest to all the other reproducible variants with moderate diagnostic predictability and suggested the importance of other potential variants for diagnostic purposes. And because there are no specific guidelines to date regarding selection of diagnostic or predictive markers, we are proposing a few probable candidates (shown in Table 5) for diagnostic/predictive genetic panel for MDD susceptibility and to predict antidepressant response.

**Table 5.** List of Replicated Variants With Single Risk Allele in MDD Susceptibility and Antidepressant Response

S. No.	Variant	Gene	Variant type	Polymorphism (Reference/alternate allele)	Calculated Risk Allele*	Sensitivity	Specificity	PPV	Regulome DB rank	Splice site	miRNA	SIFT/Polephen	Regulatory potential/conservation	Knockout mouse	Drug target	Brain expression
MDD susceptibility variants																
1	rs1801133	*MTHFR*	Missense	C/T	T	0.42	0.68	0.56	4	–	–	Deleterious/ Probably damaging	0.358/1.000	–	–	–
2	rs5443	*GNB3*	Synonymous	C/T	T	0.55	0.55	0.55	4	–	–	–	0.438/0.200	–	Y	Y
3	rs242939	*CRHR1*	Intron	C/G	G	0.13	0.93	0.66	5	–	–	–	0.238/0.000	Y	Y	Y
4	rs1006737	*CACNA1C*	Intron	G/A	A	0.31	0.78	0.59	5	–	–	–	0/0	Y	Y	Y
5	rs4880	*SOD2*	Missense	T/C	T	0.46	0.61	0.54	4	Y	–	Tolerated/benign	0.22/0	Y	–	–
6	rs6295	*5HTR1A*	Upstream	C/G	G	0.33	0.75	0.56	4	–	–	–	0/0	Y	Y	Y
7	rs1801131	*MTHFR*	Missense	A/C	C	0.33	0.71	0.53	1F	Y	–	Tolerated/benign	0.40/1.00	Y	–	–
Drug response variants																
1	STin2	*SLC6A4*	Copy number loss	L/S (12/10)	S (SSRI non response)	0.18	0.94	0.63	4	–	–	–	–	Y	Y	Y
2	rs41423247	*NR3C1*	Intron	G/C	C (SSRI non response)	0.55	0.34	0.42	2b	–	–	–	0/0	Y	Y	Y

Abbreviations: PPV, positive predicted value; SIFT, sorting intolerant from tolerant; MDD, major depressive disorder; L/S, long/short; SSRI, selective serotonin reuptake inhibitor; Y, yes (information available related to a variant within a particular category).

In addition to reproducibility with a single risk allele, these variants have clinical relevance as well. For example, out of 7, there were missense (rs1801133/*MTHFR*, rs1801131/*MTHFR*, rs4880/*SOD2*) variants including 1 SNP (rs1801133/*MTHFR*) annotated as having a deleterious/damaging effect. Two of the SNPs (rs4880/*SOD2* and rs1801131/*MTHFR*) were identified to be present at the splice site. In addition, of all the variants predicted to have a regulatory role in transcription factor binding site activity, importantly, rs1801133/*MTHFR* (score = 1F) involved eQTL function as well. The majority of genes have shown to be preferentially expressed in the brain and are known targets of antidepressant drugs. Moreover, all 7 variants from disease and 2 variants from drug response were consistently found to be associated with moderate positive predictive value. Hence, this work holds promise by highlighting biologically important markers for MDD susceptibility and antidepressant response assessment.

Even though this study is an attempt to provide a wide landscape of genetic literature, we must acknowledge the limitations as well. First and foremost, our search is limited to only MEDLINE and studies in English, so there is the possibility of non-inclusion of relevant data outside these criteria. Second, in antidepressant response search data were excluded where response is assessed based on percentage improvement and where data was not compared between responder and non-responder or remitter and non-remitter groups. However, the exclusion was according to the protocol, as we have aimed to specifically focus on studies considered dichotomous groups. Moreover, heterogeneity in antidepressant class, response status, inconsistent risk allele, and unavailability of raw data limit us to calculate the predictability of most of the replicated polymorphisms. Nevertheless, we maintained the consensus across studies by calculating the unadjusted *P* value and OR for risk allele wherever raw data (allele frequency) was provided. We found most findings were inconsistent in reporting the risk allele even in the same population across studies, which precluded us from drawing any robust conclusion by nullifying the assumption of population-specific risk allele. The reason for these inconsistencies could be addressed by small sample size and large publication bias in addition to other technical or methodological differences among studies. Further, GWAS published to date have largely not replicated the candidate gene polymorphisms conducted over the past many years, which may be attributed to the polygenic nature of the disease. Lastly, the use of sensitivity and specificity as quantitative assessment to estimate diagnostic predictability was solely based on available evidence. With the publication of more new studies, such measures may vary. Hence, our study only suggests a direction of predictability and should be used judiciously for clinical interpretations.

## CONCLUSIONS AND FUTURE DIRECTIONS

This study attempted to provide a holistic landscape of all genetic association studies related to MDD risk and antidepressant response. Despite the substantial heterogeneity in genetic association across studies, study design, and drugs administered, we identified 31 and 18 genomic regions that showed independent replication in MDD susceptibility and antidepressant response studies, respectively. Importantly, 4 variants (rs2522833/PCLO, rs2715147/PCLO, rs9416742/ BICC1, and rs999845/ BICC1) in MDD and 1 (rs6127921/BMP7) variant with antidepressant response are of high potential as they are significantly associated in both candidate and GWAS studies. Further, the variants significantly replicated in the candidate as well as GWAS studies, with consistent risk allele and moderate positive predictive values, that is, 7 (rs1801133, rs5443, rs242939, rs1006737, rs4880, rs6295, rs1801131) with MDD, and 2 (STin2, rs41423247) with antidepressant drug response, are of high interest for diagnostic and therapeutic applications after appropriate validation. Besides, this article would serve as the basis for selecting the candidate variants, and researchers can directly take up these replicated variants (31 in MDD disease and 18 in drug response) for performing future meta-analysis and functional validation. Moreover, it is also important to emphasize on remaining significant associations found in a single study. Hence, replication of these genetic associations are crucial to ascertain the significance, whether random or causal.

## Supplementary Material

pyad001_suppl_Supplementary_Tables_S1-S7
